# An *ANXA11* P93S variant dysregulates TDP‐43 and causes corticobasal syndrome

**DOI:** 10.1002/alz.13915

**Published:** 2024-06-26

**Authors:** Allison Snyder, Veronica H. Ryan, James Hawrot, Sydney Lawton, Daniel M. Ramos, Y. Andy Qi, Kory R. Johnson, Xylena Reed, Nicholas L. Johnson, Aaron W. Kollasch, Megan F. Duffy, Lawren VandeVrede, J. Nicholas Cochran, Bruce L. Miller, Camilo Toro, Bibiana Bielekova, Debora S. Marks, Jennifer S. Yokoyama, Justin Y. Kwan, Mark R. Cookson, Michael E. Ward

**Affiliations:** ^1^ Neurogenetics Branch National Institute of Neurological Disorders and Stroke National Institutes of Health Bethesda Maryland USA; ^2^ Center for Alzheimer's and Related Dementias National Institutes of Health Bethesda Maryland USA; ^3^ National Institute of General Medical Sciences National Institutes of Health Bethesda Maryland USA; ^4^ Intramural Bioinformatics Core National Institute of Neurological Disorders and Stroke National Institutes of Health Bethesda Maryland USA; ^5^ DataTecnica LLC Washington District of Columbia USA; ^6^ Department of Systems Biology Harvard Medical School Boston Massachusetts USA; ^7^ Cell Biology and Gene Expression Section Laboratory of Neurogenetics, National Institute on Aging National Institutes of Health Bethesda Maryland USA; ^8^ Memory and Aging Center Department of Neurology Weill Institute for Neurosciences University of California San Francisco San Francisco California USA; ^9^ HudsonAlpha Institute for Biotechnology Huntsville Alabama USA; ^10^ Undiagnosed Diseases Program National Human Genome Research Institute National Institutes of Health Bethesda Maryland USA; ^11^ Neuroimmunological Diseases Section National Institute of Allergy and Infectious Diseases National Institutes of Health Bethesda Maryland USA; ^12^ Broad Institute of MIT and Harvard Cambridge Massachusetts USA; ^13^ Department of Radiology and Biomedical Imaging University of California San Francisco San Francisco California USA; ^14^ National Institute of Neurological Disorders and Stroke National Institutes of Health Bethesda Maryland USA

**Keywords:** ANXA11, corticobasal syndrome, TDP‐43, variant of uncertain significance

## Abstract

**INTRODUCTION:**

Variants of uncertain significance (VUS) surged with affordable genetic testing, posing challenges for determining pathogenicity. We examine the pathogenicity of a novel VUS P93S in Annexin A11 (ANXA11) – an amyotrophic lateral sclerosis/frontotemporal dementia‐associated gene – in a corticobasal syndrome kindred. Established ANXA11 mutations cause ANXA11 aggregation, altered lysosomal‐RNA granule co‐trafficking, and transactive response DNA binding protein of 43 kDa (TDP‐43) mis‐localization.

**METHODS:**

We described the clinical presentation and explored the phenotypic diversity of ANXA11 variants. P93S's effect on ANXA11 function and TDP‐43 biology was characterized in induced pluripotent stem cell‐derived neurons alongside multiomic neuronal and microglial profiling.

**RESULTS:**

ANXA11 mutations were linked to corticobasal syndrome cases. P93S led to decreased lysosome colocalization, neuritic RNA, and nuclear TDP‐43 with cryptic exon expression. Multiomic microglial signatures implicated immune dysregulation and interferon signaling pathways.

**DISCUSSION:**

This study establishes ANXA11 P93S pathogenicity, broadens the phenotypic spectrum of ANXA11 mutations, underscores neuronal and microglial dysfunction in ANXA11 pathophysiology, and demonstrates the potential of cellular models to determine variant pathogenicity.

**Highlights:**

- *ANXA11* P93S is a pathogenic variant.
- Corticobasal syndrome is part of the *ANXA11* phenotypic spectrum.
- Hybridization chain reaction fluorescence in situ hybridization (HCR FISH) is a new tool for the detection of cryptic exons due to TDP‐43‐related loss of splicing regulation.
- Microglial ANXA11 and related immune pathways are important drivers of disease.
- Cellular models are powerful tools for adjudicating variants of uncertain significance.

## 1 BACKGROUND

With expanded access to genetic testing, the identification of novel genetic mutations and gene–disease associations has increased, but so too has the identification of variants of uncertain significance, which are the most commonly identified genetic changes and are increasing exponentially.^1^ At present, these often represent a dead end for both patients and researchers as current methods to establish causality of a variant of uncertain significance (VUS) rely on a preponderance of clinical evidence as well as imprecise in silico prediction models built from evolutionary conservation across species and magnitude of amino acid change.^2^ Therefore, cellular and in vivo models are often employed to augment informatic predictions of pathogenicity, but applications of these models poorly generalize to other genetic variants within the same disease spectrum.

Annexin A11 (ANXA11) is a disease‐associated protein that tethers RNA granules to lysosomes as they traffic in axons. The N‐terminus of ANXA11 contains a low‐complexity domain (LCD), which mediates phase transitions required for its condensation into membraneless RNA granules. Its C‐terminus harbors four calcium‐binding annexin domains, enabling regulated interactions of ANXA11 with negatively charged lysosomal membranes. Disease‐associated mutations in both the N‐terminal and C‐terminal domains of ANXA11 have been described, which alter its phase separation properties and disrupt its RNA‐lysosome tethering function.^3^, ^4^, ^5^, ^6^

The number of diseases associated with variants in ANXA11 has broadened substantially since 2006, when a genome‐wide association study first implicated it as a risk gene for autoimmune disease and sarcoid.^7^ In 2017, multiple ANXA11 mutations were found to cause genetic forms of amyotrophic lateral sclerosis (ALS).^8^ These cases exhibited both transactive response DNA binding protein of 43 kDa (TDP‐43) and ANXA11 aggregates in the central nervous system (CNS).^8^ Subsequent independent studies identified these and other ANXA11 variants in association with familial forms of frontotemporal dementia (FTD), fitting the rubric of genes causing a spectrum of disease from ALS to FTD.^9^ In 2021, ANXA11 variants were found to cause an inclusion‐body myopathy‐like syndrome affecting limb‐girdle, axial, and distal leg musculature.^10^ Clinical phenotypes within the described inclusion‐body myopathy kindreds included both ALS/FTD features and two cases of FTD, indicating that ANXA11 mutations can cause a variety of distinct phenotypes within the FTD/ALS/inclusion‐body myopathy disease spectrum.

Here we describe a family with a VUS in ANXA11 resulting in a serine in position 93 in lieu of the canonical proline, denoted by P93S, who present clinically with corticobasal syndrome (CBS), a novel clinical phenotype not previously associated with this gene. We further identified additional patients with mutations in ANXA11 and similar clinical presentations from a large neurodegenerative disease cohort, thereby establishing CBS as part of the phenotypic spectrum of ANXA11 mutations. Using induced pluripotent stem cell (iPSC)‐based models, coupled with proteomic, transcriptomic, and microscopy readouts, we show that the P93S ANXA11 variant alters key functional properties of ANXA11. Expression of the P93S ANXA11 variant causes abnormalities in TDP‐43 expression and function, which are hallmarks of ALS/FTD pathophysiology. Finally, the P93S ANXA11 variant substantially alters the transcriptome and proteome of microglia, suggesting that ANXA11 may play crucial roles in glia in addition to its known importance in neuronal biology.

## 2 METHODS

### 2.1 Clinical evaluation of the P93S kindred

Members of the P93S kindred were seen under the National Institutes of Health (NIH) Institutional Review Board‐approved protocol entitled “Investigating Complex Neurodegenerative Disorders Related to Amyotrophic Lateral Sclerosis and Frontotemporal Dementia,” and written informed consent was obtained in accordance with the Declaration of Helsinki. Enrolled participants underwent comprehensive assessments, including history and neurological examination, neuropsychological testing, and caregiver questionnaires. The motor rating was captured using an ADPM Wearable Technologies device. Electromyography and nerve conduction studies were performed. Magnetic resonance imaging (MRI) was done on a 3T scanner including sagittal three‐dimensional (3D) T1‐weighted, fat‐suppressed axial T2‐weighted, fat‐suppressed coronal T2‐weighted, axial fluid attenuated inversion recovery (FLAIR), and axial diffusion tensor images of the brain and sagittal T1‐weighted, sagittal STIR, sagittal T2‐weighted, axial T2‐weighted, axial s3D Multiple Echo Data Image Combination‐Magnetization Transfer, post‐contrast sagittal T1‐weighted, and post‐contrast axial 3D T1‐weighted images of the spine. Lumbar puncture was performed in the upright, seated position with the insertion of an atraumatic Sprotte needle in the L4/5 space and analyzed for cell counts and differential, protein, glucose, oligoclonal bands, and IgG index. Additional exploratory cerebrospinal fluid (CSF) analyses included absolute and relative quantification of CSF immune cell subsets using multicolored flow cytometry and proteomic inflammatory biomarker testing according to previously published protocols.^11^

**RESEARCH IN CONTEXT**

1. **Systematic review**: The authors reviewed the literature using PubMed sources, meeting abstracts, and presentations. While Annexin A11 has been associated with amyotrophic lateral sclerosis and frontotemporal dementia, no prior reports described corticobasal syndrome, nor has the P93S variant been reported. Moreover, corticobasal syndrome (CBS) is not commonly associated with genetic etiologies. Additionally, there is no standard workflow for the determination of the pathogenicity of novel variants of uncertain significance.
2. **Interpretation**: Our findings establish the CBS presentation of ANXA11 mutations and the pathogenicity of the P93S variant using induced pluripotent stem cell (iPSC) models, showing both loss of ANXA11 function and associated TDP‐43 pathology.
3. **Future directions**: This manuscript presents a rubric for the evaluation of VUS in genes associated with TDP‐43 pathology using iPSC models to examine gene function and a novel tool for the detection of cryptic exons related to TDP‐43‐related pathology that may be applied to many different genes.

### 2.2 Clinical spectrum of ANXA11 mutations

NIH Undiagnosed Diseases Program and the University of California San Francisco Memory and Aging Center cohorts were queried for cases harboring ANXA11 mutations using a minor allele frequency filter of 1 in 10,000 in gnomAD. Identified cases were then reviewed for diagnosis and clinical features by board‐certified neurologists (A.S., J.K., L.V.).

### 2.3 Cell culture and differentiation

The NIH Intramural research program policies were followed for the procurement and use of WTC11 line iPSC from the Coriell Institute for Medical Research cell repository, which were derived from a 30‐year‐old male without known neurological disease. Cultural procedures are described elsewhere.^12^ Briefly, iPSC were grown on tissue culture dishes precoated with Matrigel (Corning, Catalog No. 354277) in Essential 8 (E8) medium (Thermo Fisher Scientific, Catalog No. A1517001) and replaced every 1–2 days as needed. StemPro Accutase (Gibco, Catalog No. A1110501) was used for cell dissociation and passaging. Chroman‐1 (MedChemExpress, Catalog No. HY‐15392) supplementation was used to promote survival upon thawing, passaging, and other cell line modifications until cell colonies contained a minimum of five cells. The human iPSCs used in this study were previously engineered to express mouse neurogenin‐2 (mNGN2) with a doxycycline‐inducible promoter integrated in the AAVS1 and CLYBL promoter safe harbor sites for differentiation into cortical‐like neurons or transcription factors MAFB, IRF8, and CEBPA in the AAVS1 and SPI1, CEBPB, and IRF5 in the CLYBL safe harbor sites for differentiation into microglia‐like cells.

Plasmids for viral transduction into iPSCs were generated using the pLEX lentiviral vector and ligation cloning to generate fluorescently labeled vectors for the overexpression of ANXA11 based on previously published constructs.^3^ Following sequence confirmation, the plasmids were transfected into Lenti‐X human embryonic kidney cells using Lipofectamine 3000 (Invitrogen, Catalog No. L3000001). Twenty‐four hours after transfection, a viral boost reagent (Alstem, Catalog No. VB100) was added. The medium was collected after 72 h and concentrated using Lenti‐X concentrator (Takara Bio, Catalog No. 631232) overnight before being aliquoted and stored at −80°C. After dissociation and singularization using Accutase, viral aliquots were delivered to iPSCs in E8 medium with Chroman‐1. Cells were monitored for transduction efficiencies with a goal of at least 80% of cells expressing the fluorescent green construct, equally across lines. The medium was changed to E8, and iPSCs were expanded and frozen for use in differentiations and downstream experiments.

Neuronal differentiation followed previously published protocols.^12^ Briefly, cells expressing doxycycline‐inducible mNGN2 were plated on a Matrigel‐coated plate in neuronal induction medium: DMEM/F12 (Thermo Fisher Scientific, Catalog No. 11330032) supplemented with N2 supplement (Thermo Fisher Scientific, Catalog No. 17502048), non‐essential amino acids (Thermo Fisher Scientific, Catalog No. 11140050), Glutamax (Thermo Fisher Scientific, Catalog No. 35050061), 2 µg/mL doxycycline (Sigma‐Aldrich, Catalog No. D9891), and 50 nM Chroman‐1 on day 0. Fresh induction medium without Chroman‐1 was added after PBS washes on days 1 and 2. On day 3, cells were dissociated using Accutase, counted, and replated onto poly‐L‐ornithine (PLO)‐coated plates in neuronal maturation medium containing 2 µg/mL doxycycline. On day 4, the medium was changed after a PBS wash. Thereafter, a half‐medium change was conducted twice weekly. All neuron experiments were conducted using day 15 neurons unless otherwise specified.

Microglial differentiation followed previously published protocols.^13^ Briefly, cells expressing the six transcription factors were plated on a dual Matrigel‐ and PLO‐coated tissue culture dish in E8 medium with doxycycline and Chroman‐1 on day 0. On day 2, the medium was changed to Advanced DMEM/F12 (Thermo Fisher Scientific, Catalog No. 12634010) with 2 µg/mL doxycycline, GlutaMAX (Thermo Fisher Scientific, Catalog No. 35050061), 100 ng/mL Human IL‐34 (Peprotech, Catalog No. 200‐34), and 10 ng/mL Human GM‐CSF (Peprotech, Catalog No. 300‐03) after PBS washes. On day 4, medium was changed with the addition of 50 ng/mL Human M‐CSF (Peprotech, Catalog No. 300‐25), 50 ng/mL Human TGF‐β1 (Peprotech, Catalog No. 100‐21C), 50 ng/mL Mevalonolactone (Sigma‐Aldrich, Catalog No. M4667‐1G), and antibiotic/antimycotic (Gibco, Catalog No. 15240‐062). Thereafter, half‐medium changes were conducted twice weekly. All microglial experiments were performed on day 15 unless otherwise specified.

### 2.4 Lysosome colocalization

WTC11 iPSCs with the mNGN2 construct overexpressing wild‐type (WT) and P93S mutant ANXA11 were transduced with a lentiviral vector containing LAMP1 fluorescently labeled with mApple as described in cell culture methods above. Following transduction, iPSCs were differentiated into iPSC‐derived neurons as spheroids. For spheroid differentiation, 10,000 iPSCs were resuspended in 20 µL neuronal induction medium per sphere after splitting iPSCs with Accutase. A total of 20 µL of the iPSC suspension was added to a well of an ultra‐low‐attachment round‐bottom 384‐well plate (Corning) coated with anti‐adherence solution (STEMCELL Technologies, Catalog No. 07010) for 1 h and washed with PBS three times. Cells were left to sit for 5 min before centrifuging at 150 relative centrifugal force for 2 min to promote cell aggregation. The next day, 60 µL neuronal induction medium was added. On the the third day after addition of doxycycline, spheres were pipetted up using a wide‐bore, low‐attachment pipette tip and plated on an eight‐chamber glass‐bottom slide (Ibidi) coated with poly‐L‐ornithine, followed by a 2‐h coating with 15 µg/mL laminin and prepped with 250 uL neuronal maturation medium. Spheroids were allowed to grow for four more days with a full medium change the day after replating and a half‐medium change every 3 days thereafter. On day 7 after doxycycline, spheroids were imaged using a Nikon spinning‐disk confocal microscope equipped with a 60× water immersion objective. Live cell imaging was focused on regions of the well with clear growth cones and relatively sparse axons to reduce the number of intersecting axons in the captured image area. Eight images were captured per well with four replicates per condition. NIS‐Elements General Analysis 3 (GA3) was used to quantify red and green puncta corresponding to lysosomes and ANXA11 granules, respectively. Total counts of lysosomes colocalized with ANXA11 were calculated as well as mean counts per well. Data were assessed for normality using a Shapiro–Wilk test, and an unpaired *t* test was applied to well means to determine significance. Data were visualized in GraphPad Prism version 9.5.1.

### 2.5 Quantification of neuritic RNA

RNAscope Assay (Advanced Cell Diagnostics) was used to assess the delivery and distribution of RNA in iPSC‐derived neurons according to the manufacturer's protocol.^14^ Briefly, iPSC‐derived neurons overexpressing WT and P93S mutant ANXA11 were plated on an eight‐chamber glass‐bottom slide (Ibidi) at a density of 12,000 cells per well in 250 µL medium. On day 15 after doxycycline, cells were fixed with 4% paraformaldehyde for 10 min at room temperature, then washed three times with PBS. Cells were then sequentially dehydrated in increasing concentrations of ethanol and frozen at −20°C overnight. The following day cells were rehydrated before treatment with protease. The cells were probed for beta‐actin in a HybEZ oven at 40°C for 2 h. Cells were incubated with Amp1‐FL, Amp2‐FL, Amp3‐FL, and Amp4‐FL solutions sequentially prior to Hoechst nuclear counterstaining. Cells were imaged immediately for the identification of beta‐actin RNA puncta. The following day, cells were incubated with anti‐tau (R&D AF3494) and anti‐H4A3 (DSHB AB 2296838) antibodies in PBS at a 1:1000 dilution overnight at 4°C. The next day, cells were washed three times with PBS before being incubated with anti‐goat and anti‐rabbit secondary antibodies (Biotium, Catalog No. 20016 and 20098) for 30 min at room temperature and reimaged. Imaging was performed on the Nikon spinning‐disk confocal microscope (Nikon Eclipse T1) using a 60× water immersion objective lens. Eight images were captured per well with eight replicates per condition. NIS‐Elements GA3 was used to quantify RNA puncta. Total neuritic RNA puncta per total RNA puncta were calculated as well as means per well. Data were assessed for normality using the Shapiro–Wilk test, and unpaired *t* test was applied to well means to determine significance. Data were visualized in GraphPad Prism version 9.5.1.

### 2.6 TDP‐43 immunocytochemistry

Immunocytochemistry was performed on 96‐well plates (Perkin Elmer) or µ‐Slide glass‐bottom slides (Ibidi, Catalog No. 80826). On day 15 after differentiation, cells were fixed using 4% paraformaldehyde for 15 min at room temperature. Following three PBS washes, cells were permeabilized using 0.1% Triton‐X100 for 10 min at room temperature. Cells were then blocked in 2% BSA for 60 min. Primary antibody incubation was performed overnight at 4°C at a 1:500 concentration of anti‐TDP‐43 antibody (Abcam ab254166). Immunofluorescence was detected with fluorochrome‐conjugated secondary antibodies CF640 donkey anti‐mouse (1:1000) for detection of TDP‐43. Finally, nuclei were counterstained with Hoechst (2 µg/mL) (Thermo Fisher Scientific, Catalog No. 62248). Images were acquired on an inverted Nikon spinning‐disk confocal microscope (Nikon Eclipse T1) using a 60× 1.40 NA oil‐immersion objective. Twelve images were captured per well with four replicates per condition. Quantification of nuclear TDP signal was performed using CellProfiler. Total mean intensities of nuclear TDP‐43 signal were calculated, as were means per well. Data were tested for normality using Shapiro–Wilk test, and an unpaired *t* test was applied to well means to determine significance. Data were visualized in GraphPad Prism version 9.5.1.

### 2.7 Detection of cryptic exons

Hybridization chain reaction fluorescence in situ hybridization (HCR FISH) custom probes were designed using the Molecular Instruments Custom Probe Design Tool to target native and cryptic exons in two genes known to be associated with TDP‐43 loss of function: UNC13A and STMN2 (Supplementary Material). Ability to detect cryptic RNA was first tested in iPSC‐derived neurons expressing a catalytically dead Cas9 fused to a KRAB transcriptional repression domain to allow inhibition of gene transcription with control non‐targeting guide and a guide to knockdown (KD) TDP‐43 expression on day 7 after doxycycline. HCR FISH was performed according to the manufacturer's protocol.^15^ Briefly, iPSC‐derived neurons overexpressing WT and P93S mutant ANXA11 were plated on a 384‐well plate (Corning) at a density of 3000 cells per well in 100 µL medium. On day 15 after doxycycline, cells were fixed with 4% paraformaldehyde for 10 min at room temperature, then washed three times with PBS. Fixed cells were permeabilized with 70% ethanol overnight at −20°C. Ethanol was removed the following day, and cells were washed twice with 2× SSC buffer (Invitrogen, Catalog No. 15557044). Before addition of the probe solutions, cells were incubated in a warm probe hybridization buffer for 30 min at 37°C. Probe solutions were prepared with 0.8 pmol of each probe set in a probe hybridization buffer. Cells in probe solutions were hybridized overnight at 37°C. The following day, cells were washed four times with a warm probe wash buffer. Cells were then washed twice with 5x SSCT at room temperature. Samples were amplified for 30 min at room temperature in an amplification buffer while hairpin solutions were prepared. A total of 12 pmol of each hairpin one and two were heated to 95°C for 90 s and then cooled to room temperature without exposure to light for 30 min. Cooled hairpins were added together in an amplification buffer at room temperature before being added to the cells. Samples were incubated at room temperature overnight without exposure to light. The next day, hairpin solutions were removed, and cells were washed five times with 5x SSC‐Tween 20. Cells were incubated with Hoechst in PBS for nuclear counterstaining at a 1:10,000 dilution for 5 min at room temperature. Cells were washed three times with PBS and stored at 4°C until being imaged. Cells were imaged with a Nikon spinning‐disk confocal microscope on a 60× water immersion objective lens using a random imaging job with the nucleus as the plane of focus. NIS‐Elements GA3 was used to quantify cryptic exons. Total cryptic exons per cell were calculated, as were means per well. Data were assessed for normality using a Shapiro–Wilk test. To determine significance, an unpaired *t* test was applied to data passing tests of normality, and a Mann‐Whitney test was applied to data not passing tests of normality. Data were visualized in GraphPad Prism version 9.5.1.

### 2.8 Single‐cell RNA sequencing

iPSC‐derived neurons and microglia on day 15 after differentiation were washed with PBS once and then washed twice with PBS + 0.5 mM EDTA before the addition of 1 mL of papain at 10 units/mL (Worthington) in TrypLE. Cells were incubated for 5 min at 37°C until cells started to physically detach. Enzyme mixture was aspirated, and cells were resuspended in a trituration medium (BrainPhys medium supplemented with Chroman‐1 and 33 µg/mL DNase I [Worthington]) using a p1000 pipette tip until single cells were visible under light microscopy. Cells were collected and centrifuged at 200 × *g* for 5 min at room temperature. The supernatant was aspirated and cells were resuspended in a trituration medium with ovomucoid papain inhibitor at 10 mg/mL (Worthington). Cells were centrifuged at 200 × *g* for 5 min at room temperature. Cells were then resuspended in BrainPhys medium and counted. Cells were then washed three times with PBS + 0.04% BSA using a wide‐bore tip p1000 pipette. Cell solutions were counted using an automated cell counter (Countess II). The final cell solution was recounted and diluted to a target concentration of 1000 cells/µL and placed on ice.

Single cells were isolated using the Chromium Connect platform, where 5000 single cells were targeted for capture from each sample. Single‐cell expression libraries were constructed using the Chromium Next GEM Automated Single Cell 3′ Library and Gel Bead Kit version 3.1 and the Chip G Automated single‐cell kit (10x Genomics). Libraries were pooled and sequencing was completed on an Illumina NextSeq 550 system using a NextSeq 150 Cycle Hi‐Output version 2.5 kit (Illumina, Catalog No. 20024907), generating a total of 400 million reads for an estimated sequencing depth of 40,000 reads per cell.

Raw sequencing data were aligned using bcl2fastq version 2.20.0, and count tables were generated using Cell Ranger software version 7.0.0 (10x Genomics). Normalization, integration, and clustering analysis of these two scRNAseq datasets were completed using the Seurat package (version 4.3.0) in R as previously described.^16^, ^17^ Data were filtered to a minimum of 800 unique molecular identifiers (UMIs) per cell and 200 genes per cell. Cells with more than 6000 genes or greater than 10% mitochondrial reads were excluded, and a minimum 0.8 ratio of the base 10 logs of genes per cell and UMIs was required per cell. Uniform Manifold Approximation and Projection (UMAP) clustering was performed using the Louvain algorithm at a resolution of 0.2 and included 32 principal components. The neuronal cluster was identified by expression of TUBB3 and DCX; however, PHOX2B‐positive neurons were not included in pseudobulk differential expression analysis. The microglial cluster was characterized by the expression of APOE and AIF1. Pseudobulk analysis was completed using DESeq2 version 1.38.0, which uses a Wald test to compare groups and applies the Benjamini–Hochberg post hoc correction.^18^ The linear model was used to account for batch effects in the data. The ashr shrinkage algorithm was applied to penalize high log fold changes of minimally expressed transcripts. Transcripts surviving correction for multiple comparisons with adjusted *p* values < .05 were entered into ShinyGO version 0.77 for identification of Gene Ontology (GO) terms and pathways.^19^ Cytoscape was used for the visualization of interaction networks.^20^

### 2.9 Mass spectrometry‐based proteomics

Sample preparation for proteomics was performed according to previously published protocols and included an automated pipeline for protein assay, capture, and digestion.^21^, ^22^ Briefly, iPSC‐derived neurons and microglia were harvested on day 15 from six‐well plates with six replicates per condition. Cells were washed with ice‐cold PBS prior to collection using a high‐percentage detergent lysis buffer (50 mM HEPES, 50 mM NaCl, 5 mM EDTA 1% SDS, 1% Triton X‐100, 1% NP‐40, 1% Tween 20, 1% deoxycholate, and 1% glycerol) supplemented with complete protease inhibitor cocktail at 1 tablet/10 mL ratio. The cell lysate was reduced by 10 mM dithiothreitol for 30 min at 60°C and alkylated using 20 mM iodoacetamide for 30 min in the dark at room temperature. The denatured proteins were captured by hydrophilic magnetic beads. Tryptic on‐bead digestion was conducted for 16 h at 37°C. Tryptic peptides were dehydrated and resuspended in a 2% acetonitrile/0.4% trifluoroacetic acid solution and normalized to a concentration of 0.2 mg/mL for liquid chromatography‐mass spectrometry (LC‐MS) analysis.

For the LC‐MS analysis, we employed a direct‐data‐independent acquisition (dDIA) single‐shot approach. Briefly, sample peptides were separated on a nano LC and subsequently analyzed on an Orbitrap Eclipse MS. A linear 120‐min LC gradient with 2% to 35% solvent B (0.1% formic acid, 5% dimethyl sulfoxide in acetonitrile) was used on 75 µm by 500 mm LC column with 2 µm C_18_ particle (Thermo Fisher Scientific, Catalog No. ES903). The MS1 scan was set at 12,000 resolutions with an auto‐injection time; the MS2 scan isolation windows were set to 8 m/z (400 to 1000 m/z range), 3‐s cycle time, and 30,000 resolution. For protein annotation, MS raw files were database searched using a dDIA approach in Spectronaut (version 14.1, Biognosys) against a human proteome reference containing 20,586 reviewed protein entries (Uniprot‐Human‐Proteome_UP000005640). The raw intensity of quantified proteins was medium normalized across all samples from the same condition.

Normalized peptide abundances were pedestalled by two then base 2 log‐transformed. Cyclic loess normalization was applied to correct for differences in distribution. Mean abundance per protein was modeled by the coefficient of variation observed per protein per condition. Modeling non‐linear fits identified 9.5 as the value at which linearity was lost and thus applied as the filter. For differential testing, the Welch‐modified *t* test was used. For differential selection, the criteria used included a corrected *p* value < .05 and a linear fold‐change magnitude > 1.5. For correction, the Benjamini–Hochberg false discovery rate (FDR) multiple comparison correction procedure was used. All peptides were visually depicted by a volcano plot generated by GraphPad Prism version 9.5.1. Peptides meeting the differential selection criteria listed above were depicted in color on the volcano plot, with red corresponding to upregulated and blue corresponding to downregulated peptides. Differentially selected peptides meeting the above criteria were entered into Enrichr for GO enrichment analysis.^23^, ^24^, ^25^

## 3 RESULTS

### 3.1 Clinical evaluation of P93S kindred

**FIGURE 1:**
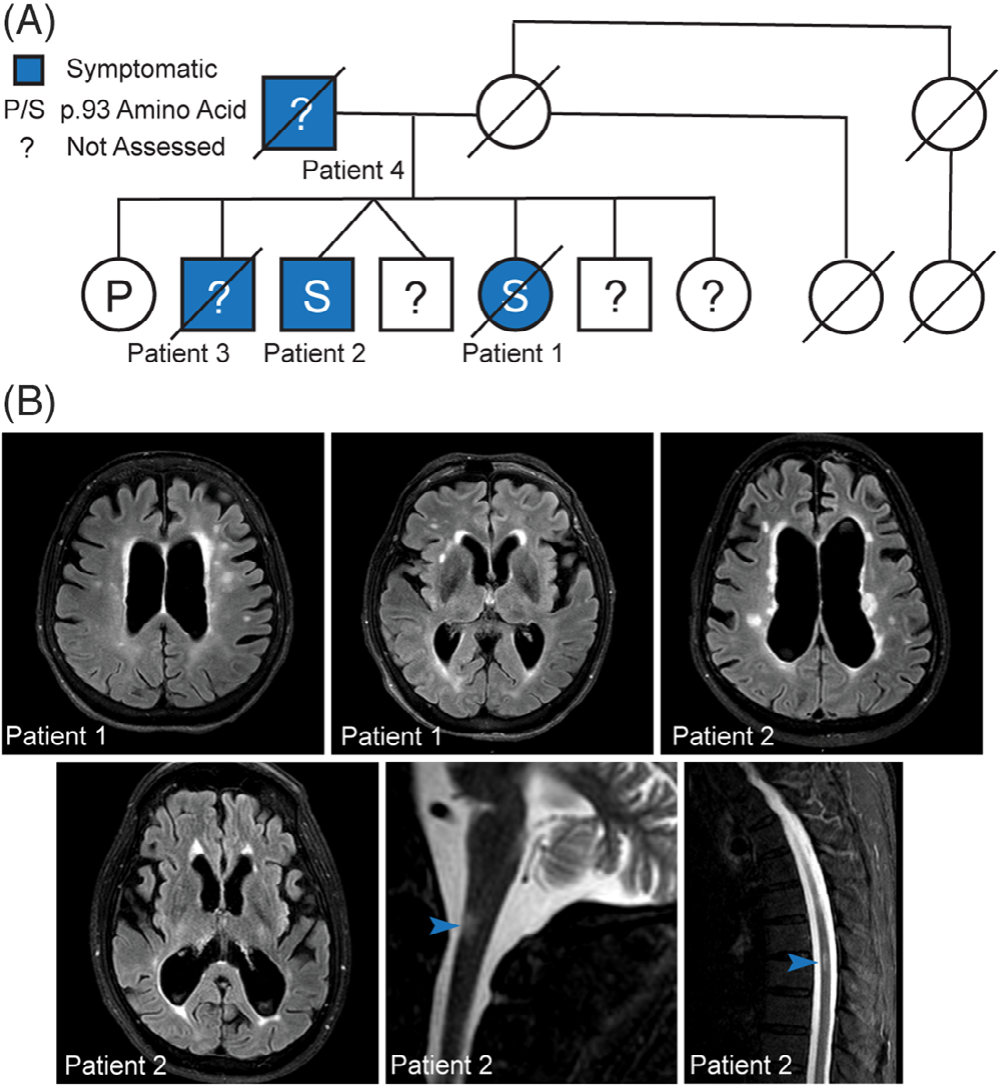
P93S kindred. (A) Pedigree of P93S family. Genetic testing is available from three individuals including two symptomatic and one asymptomatic; the position 93 amino acid is indicated by a letter, where P indicates proline and S indicates serine; unavailable testing is indicated by a question mark. Symptomatic individuals (blue) include patients 1, 2, 3, and 4. Diagonal lines indicate individual is deceased. Circles indicate female, squares male. (B) Representative MRI findings. T2‐weighted FLAIR MRI images from patients 1 and 2 demonstrating central‐predominant atrophy in a frontoparietal distribution and prominent white matter hyperintensities in the cerebral cortex and white matter hyperintensities on spinal cord images from patient 2, indicated by arrowheads.

We identified four individuals across two generations with a corticobasal‐like syndrome harboring a novel variant in the *ANXA11* gene. Whole‐exome sequencing of the proband, one affected sibling, and one unaffected sibling revealed a variant of uncertain significance in ANXA11 c.277C > T p.P93S (rs753295755) in exon 4 that segregated with disease (Figure 1A). This P93S missense variant resulting in a serine at position 93 occurs in the intrinsically disordered LCD at the N‐terminus of the protein. All affected family members present with a slowly progressive asymmetric corticobasal‐like syndrome and white matter abnormalities on MRI (Figure 1B). Both the proband and her brother suffered from chronic pain as well. CSF analysis of the proband and her brother revealed no significant abnormalities in the absolute and relative numbers of CSF cells of adaptive immunity. However, both patients had elevated proportions of innate lymphoid cells, either in CSF (proband) or blood (brother), and the proband also had a higher proportion of CSF granulocytes. Both patients had highly elevated CSF neurofilament light, a marker of neuro‐axonal injury, and CHIT3L1 a marker associated with intrathecal activation of innate immunity, expressed mainly in astrocytes and macrophages/microglia. Both subjects also had slightly elevated CSF IgG levels, but only the proband had an increased IgG index. Consistent with normal levels of CSF T cells, CSF levels of T‐cell activation marker sCD27 were also normal (Table 1; Supplementary Material).

**TABLE 1:** ANXA11 VUS cases and clinical presentations.

Patient no.	Mutation	Age	First symptom	Clinical features	MRI findings	CSF
1	P93S	60 to 77	Right leg spasticity	Bradykinesia, asymmetric spasticity, dystonia, apraxia, dysphagia, dysarthria	WMH Central predominant atrophy Corpus callosum thinning	Elevated protein, elevated IgG and IgG index, negative oligoclonal bands
2	P93S	50 to 79^a^	Right leg spasticity	Apraxia, dystonia, cortical sensory loss, alien limb	WMH Spinal WMH Central predominant atrophy Corpus callosum thinning	Elevated protein, elevated IgG, negative oligoclonal bands
3	P93S^b^	Unknown to 49	Lower extremity dysfunction	Tremor, dysarthria, dystonia	WMH	N/A
4	P93S^b^	Unknown to 60	Lower extremity dysfunction	Spasticity, dysarthria, dysphagia, dystonia	N/A	N/A
5	G189E	51 to 66	Lower extremity spasticity	Dysarthria, dysphagia	WMH	Elevated IgG, matched serum, and CSF oligoclonal bands
6	Y103H	59	Left arm apraxia	Corticobasal syndrome	Central predominant atrophy, corpus callosum thinning	N/A
7	R404W	N/A	N/A	Corticobasal syndrome	WMH, central predominant atrophy, corpus callosum thinning	N/A
8	S55L	N/A	Behavioral variant frontotemporal dementia	Parkinsonism	Central predominant atrophy	N/A
9	S55L^b^	N/A	Spasticity	Cortical sensory loss, dysphagia	N/A	N/A

Abbreviations: WMH, white matter hyperintensities; FLAIR, fluid attenuated inversion recovery; N/A, not available.

^a^ Still alive.

^b^ Presumed variant carrier; no genetic testing available.

### 3.2 Clinical spectrum of ANXA11 mutations

To further characterize the spectrum of clinical phenotypes and ANXA11 mutations, we queried neurodegenerative disorder clinics for additional cases of ANXA11 mutations. No additional cases of the P93S variant were identified; however, we identified three additional cases of CBS in carriers of previously identified ANXA11 mutations or variants of uncertain significance (Table 1), along with the previously described clinical phenotypes such as behavioral variant FTD, ALS/FTD, and ALS cases (Table 1; Supplementary Material).

In general, this clinical presentation was typified by slowly progressive disease, in contrast with ANXA11‐associated ALS cases. CNS imaging studies revealed white matter atrophy in a frontoparietal distribution with or without T2 FLAIR hyperintensities. Taken together, this kindred and other ANXA11 mutation carrier cases expand the known clinical spectrum of mutations in ANXA11 to include a CBS presentation.

### 3.3 Computational assessment of ANXA11 P93S variant

**FIGURE 2:**
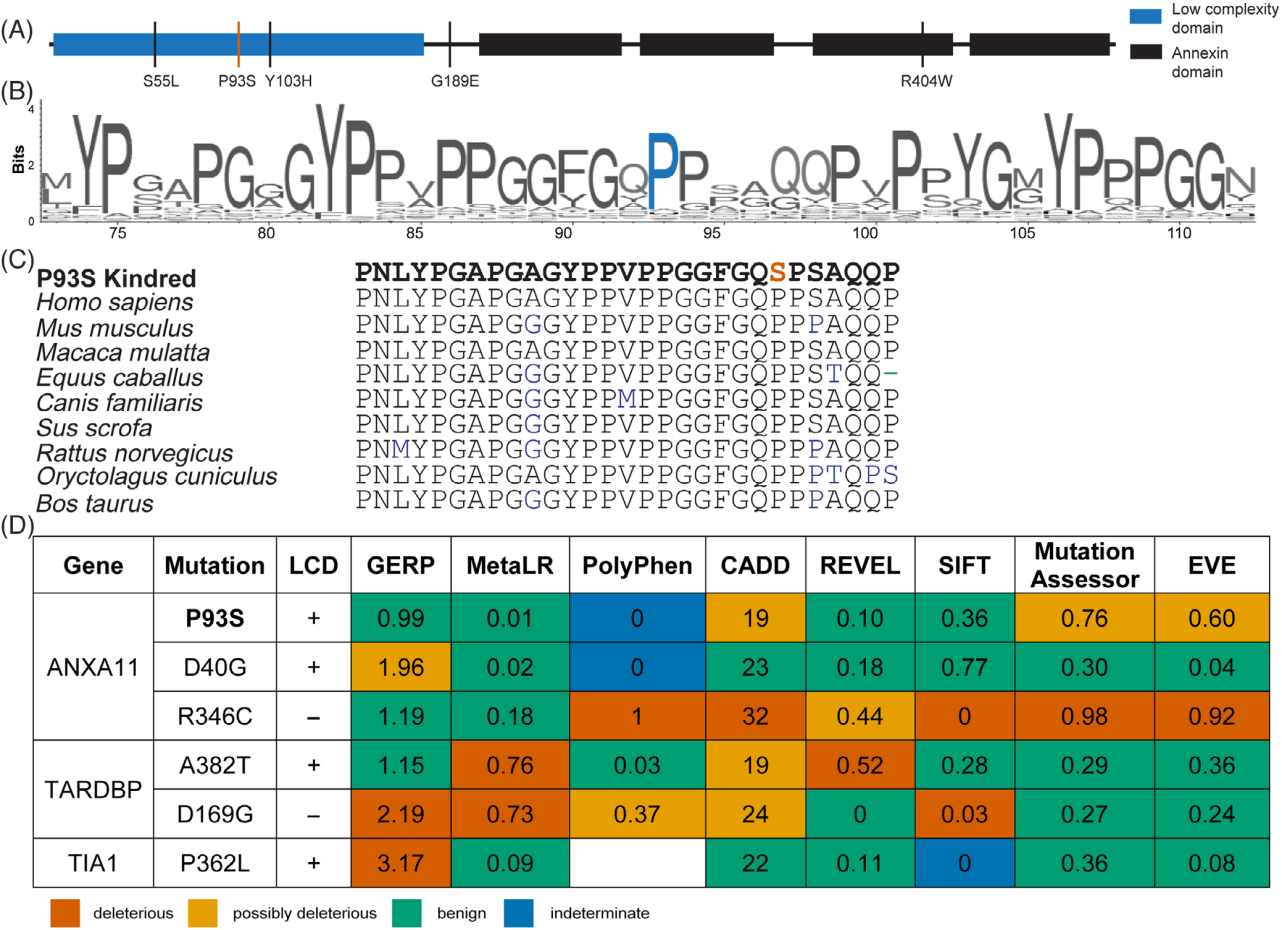
ANXA11 structure. (A) Schematic of ANXA11. Low‐complexity domain (LCD) is shown in blue, annexin domains in black. The P93S variant is identified by a red line; remaining variants/mutations are indicated by black lines. (B and C) ANXA11 sequence conservation. VarSite illustration of sequence conservation with proline in position 93 indicated in blue (B) and sequence conservation of annexin A11 across vertebrate species (C) with the P93S variant indicated in red. (D) Poor performance of in silico variant prediction models for mutations in LCDs. P93S VUS with other known pathogenic mutations in common amyotrophic lateral sclerosis/frontotemporal dementia‐associated genes with LCDs is shown. The predictions of pathogenicity by color: predicted benign in green, moderate in orange, pathogenic in red, and indeterminate in blue. GERP score considered deleterious when >2. MetaLR score ranges from 0 benign to 1 deleterious. PolyPhen score considered deleterious when >0.446. CADD score considered deleterious when >30. REVEL score ranges from 0 benign to 1 deleterious. SIFT score considered deleterious when <0.05. Mutation assessor score ranges from 0 benign to 1 deleterious. EVE score considered potentially deleterious when >0.5 and deleterious when >0.7.

The P93S VUS is located within the LCD, a region of ANXA11 in which previously identified ALS/FTD mutations reside (Figure 2A). The proline at position 93 of ANXA11 is highly conserved in land mammals, suggesting a potential physiological importance to protein function (Figure 2B,C). However, further characterization of the potential pathogenicity of the P93S variant using a set of in silico models was inconclusive (Figure 2D), with only some models suggesting even moderate pathogenicity (eg, MutationAssesor, EVE, CADD), while most others were suggestive of a benign change.^26^, ^27^, ^28^, ^29^, ^30^, ^31^, ^32^, ^33^, ^34^ The variant is extremely rare in population databases, with the identification of only one carrier of unknown age that overlaps between gnomAD version 3.1.2 (76,156 genomes) and TOPMed Bravo freeze 10 (150,899 genomes) and one carrier, which may be the same individual, age 35 to 40 in exomes (125,748 total) from gnomAD version 2.1.1. This rarity is consistent with pathogenicity as many dominant and established pathogenic neurodegeneration‐associated variants are present in population databases with even higher counts, for example, PSEN1 A79V (five counts in gnomAD version 3.1.2) or MAPT R406W (four counts in gnomAD version 2.1.1). Collectively, these findings hint at the possibility that P93S is pathogenic, but in isolation they are insufficient to assign definitive causality to this novel variant, especially given the pathologic heterogeneity of CBS.^35^

### 3.4 P93S variant alters core functions of ANXA11

**FIGURE 3:**
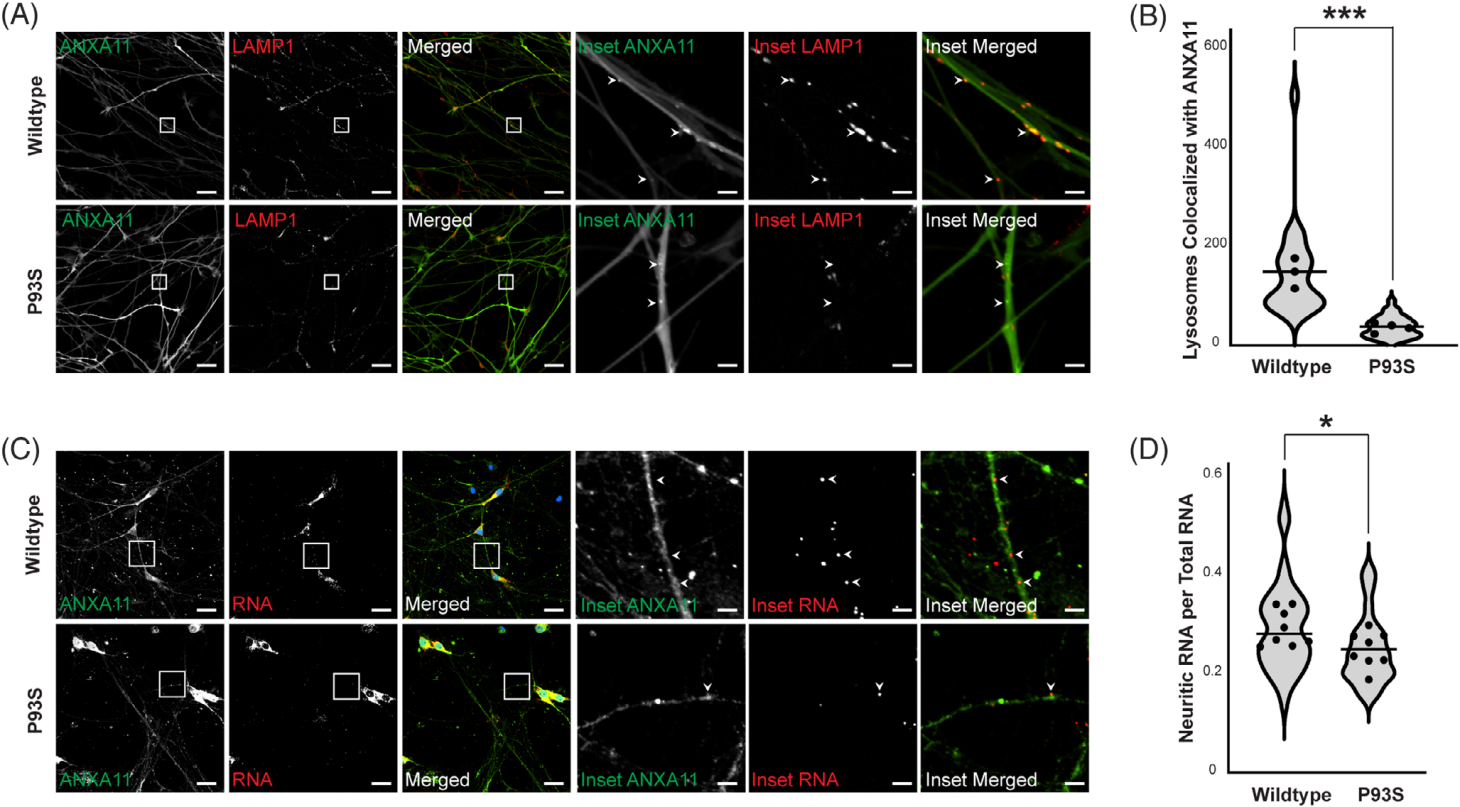
Functional impact of P93S variant. (A and B) Decreased colocalization of lysosomes in mutant ANXA11 neurons. (A) Representative images of iPSC‐derived neurons expressing WT and mutant ANXA11 (green) and LAMP1 lysosomal marker (red) showing colocalization of lysosomes with ANXA11 puncta indicated by arrowheads; scale bar = 25 µm, inset scale bar = 4 µm. (B) Quantification of number of lysosomes with ANXA11, well mean indicated by dot, horizontal line indicates median, *p* = .0009. (C and D) Decreased neuritic RNA in mutant ANXA11 neurons. (C) Representative images of fixed iPSC‐derived neurons expressing WT and mutant ANXA11 with in situ hybridization probes for β‐actin RNA using RNAscope to identify neuritic RNA indicated by arrowheads, scale bar = 25 µm, inset scale bar = 4 µm. (D) Quantification of proportion of neuritic RNA over total RNA, well mean indicated by dot, horizontal line indicates median, *p* = .03.

Given the contradictory previously discussed in silico predictions of the P93S variant, we turned to cellular models to characterize its effects on protein function. Prior studies showed that ANXA11 disease‐associated mutations could disrupt the interaction of ANXA11 with lysosomes.^3^ Therefore, we tested whether the P93S variant altered ANXA11 colocalization with lysosomes by imaging iPSC‐derived human neurons infected by lentiviruses expressing either WT or P93S ANXA11 and the LAMP1 lysosome marker (Figure 3A). We calculated the number of lysosomes colocalizing with ANXA11 granules and found that the P93S mutation caused a 75% decrease in the association of the ANXA11 granules with lysosomes (mean = 143.8 vs 35.34, *p *= .0009; Figure 3B; Figure S1A,B). ANXA11 tethers RNA granules to lysosomes during axonal transport. We examined whether the P93S variant altered axonal RNA trafficking in iPSC‐derived neurons using RNAscope, an RNA in situ hybridization technique (Figure 3C). Expression of the P93S variant reduced axonal beta‐actin RNA abundance compared to WT ANXA11 by 15% (mean = 0.2816 vs 0.2398, *p *= .03; Figure 3D). These findings indicate that P93S alters the ability of ANXA11 to interact with lysosomes, consequently impeding delivery of RNA to distal neuritic compartments.

### 3.5 P93S variant causes nuclear clearance and TDP‐43 dysfunction

**FIGURE 4:**
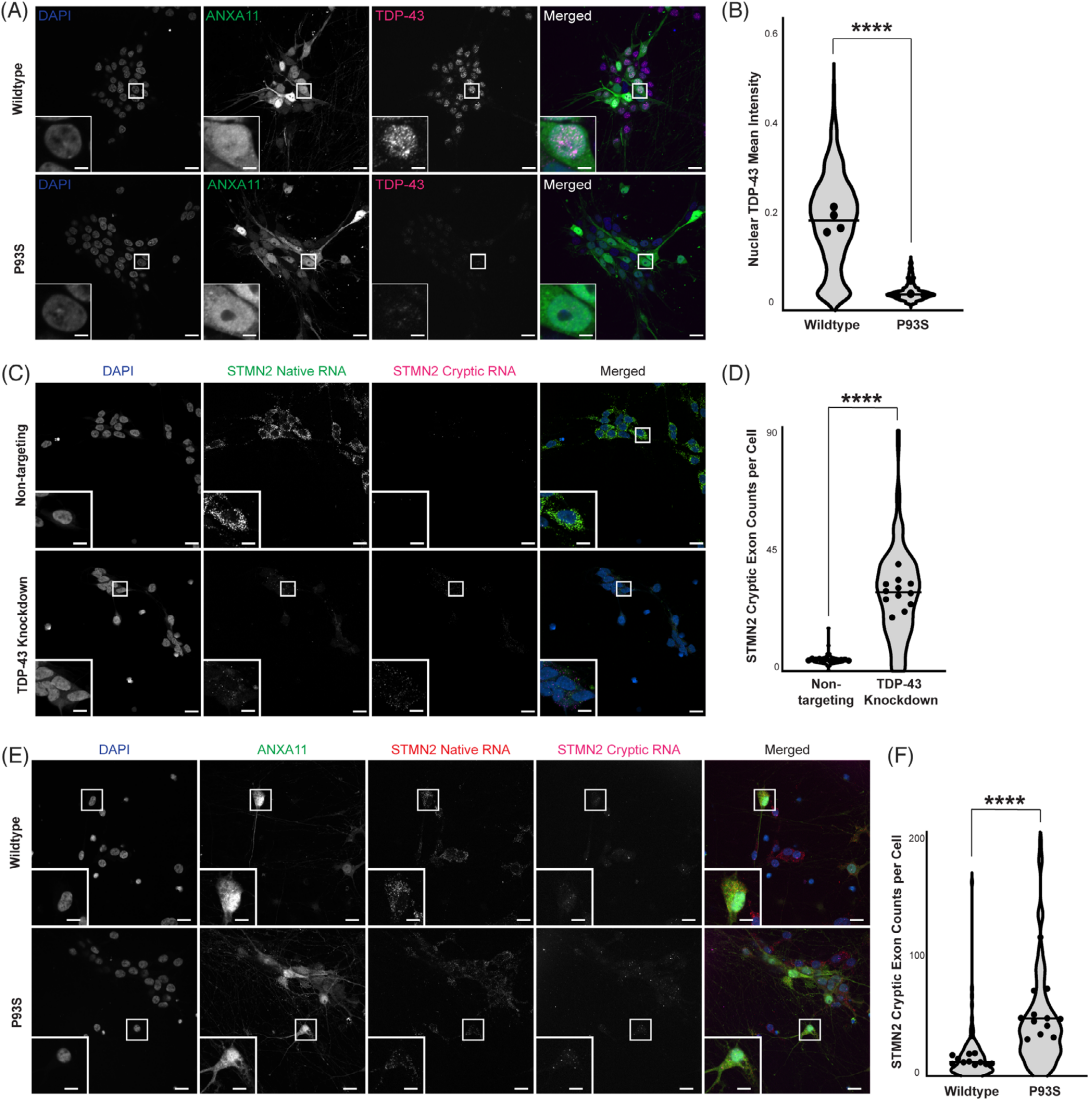
Decreased nuclear TDP‐43 and formation of cryptic exons. (A and B) Decreased nuclear TDP‐43 in mutant ANXA11 neurons. (A) Representative images of fixed iPSC‐derived neurons expressing wild‐type (WT) and mutant ANXA11 (green) stained with TDP‐43 (magenta) and Hoechst nuclear counterstaining (blue) demonstrating nuclear clearing of TDP‐43 in mutant ANXA11 cells; scale bar = 25 µm, inset scale bar = 1.75 µm. (B) Quantification of mean TDP‐43 intensity in WT compared to mutant ANXA11, well mean indicated by dot, horizontal line indicates median, *p* < .0001. (C and D) Detection of STMN2 cryptic exon formation in TDP‐43 KD neurons. (C) Representative images of fixed iPSC‐derived CRISPRi neurons with control non‐targeting and TDP‐43 knockdown guides demonstrating detection of native STMN2 RNA (green) and cryptic RNA (magenta) using HCR FISH probes with Hoechst nuclear counterstaining (blue), scale bar = 25 µm, inset scale bar = 4 µm. (D) Quantification of cryptic exon counts per cell in non‐targeting and TDP‐43 KD cells for STMN2, well mean indicated by dot, horizontal line indicates median, Mann–Whitney *p* < .0001. (E and F) Increased STMN2 cryptic exon formation in mutant ANXA11 neurons. (E) Representative images of fixed iPSC‐derived neurons expressing WT and mutant ANXA11 (green) with HCR FISH probes for native STMN2 RNA (red) and cryptic RNA (magenta) with Hoechst nuclear counterstaining (blue); scale bar = 25 µm, inset scale bar = 4 µm. (F) Quantification of cryptic exon counts per cell in WT and mutant ANXA11 cells for STMN2, well mean indicated by dot, horizontal line indicates median, *p* <0.0001.

Patients with ALS/FTD due to ANXA11 mutations develop hallmark pathological features of TDP‐43 mislocalization, including its loss in the nucleus and aggregation in the cytoplasm.^8^, ^36^ We evaluated our P93S and WT ANXA11‐expressing iPSC‐derived neurons for evidence of TDP‐43‐related changes using immunocytochemistry (Figure 4A). Expression of P93S ANXA11 resulted in substantial loss of nuclear TDP‐43 staining in iPSC‐derived neurons (mean = 0.185 vs 0.386, *p *< .0001; Figure 4B). Through direct interactions with intronic regions of pre‐mRNAs, TDP‐43 functions as a splicing repressor, and functional loss of nuclear TDP‐43 causes abnormal splicing products termed cryptic exons.^37^ Two of the most‐characterized cryptic exons related to TDP‐43 loss of function include those found in STMN2 and UNC13A mRNA transcripts (Figure 4C,D; Figure S2A,B).^38^, ^39^, ^40^, ^41^ We developed a new HCR FISH method to identify and quantify cryptic exons in STMN2 and UNC13A in fixed iPSC‐derived neurons. Using our HCR FISH assay, we quantified cryptic exon abundance in iPSC‐derived neurons expressing WT or P93S ANXA11. Expression of P93S ANXA11 increased STMN2 cryptic exon expression per cell by 3.5‐fold (mean = 14.65 vs. 52.55, *p *< .0001; Figure 4E,F) and the ratio of cryptic UNC13A transcripts to native per cell by 37% (mean = 0.1554 vs 0.2131, *p *= .04; Figure S2C,D). These results indicate that expression of P93S ANXA11 in human neurons results in histological and functional loss of nuclear TDP‐43, consistent with other previously described ANXA11 mutations associated with ALS/FTD.

### 3.6 P93S variant alters transcriptome and proteome of neurons and microglia

**FIGURE 5:**
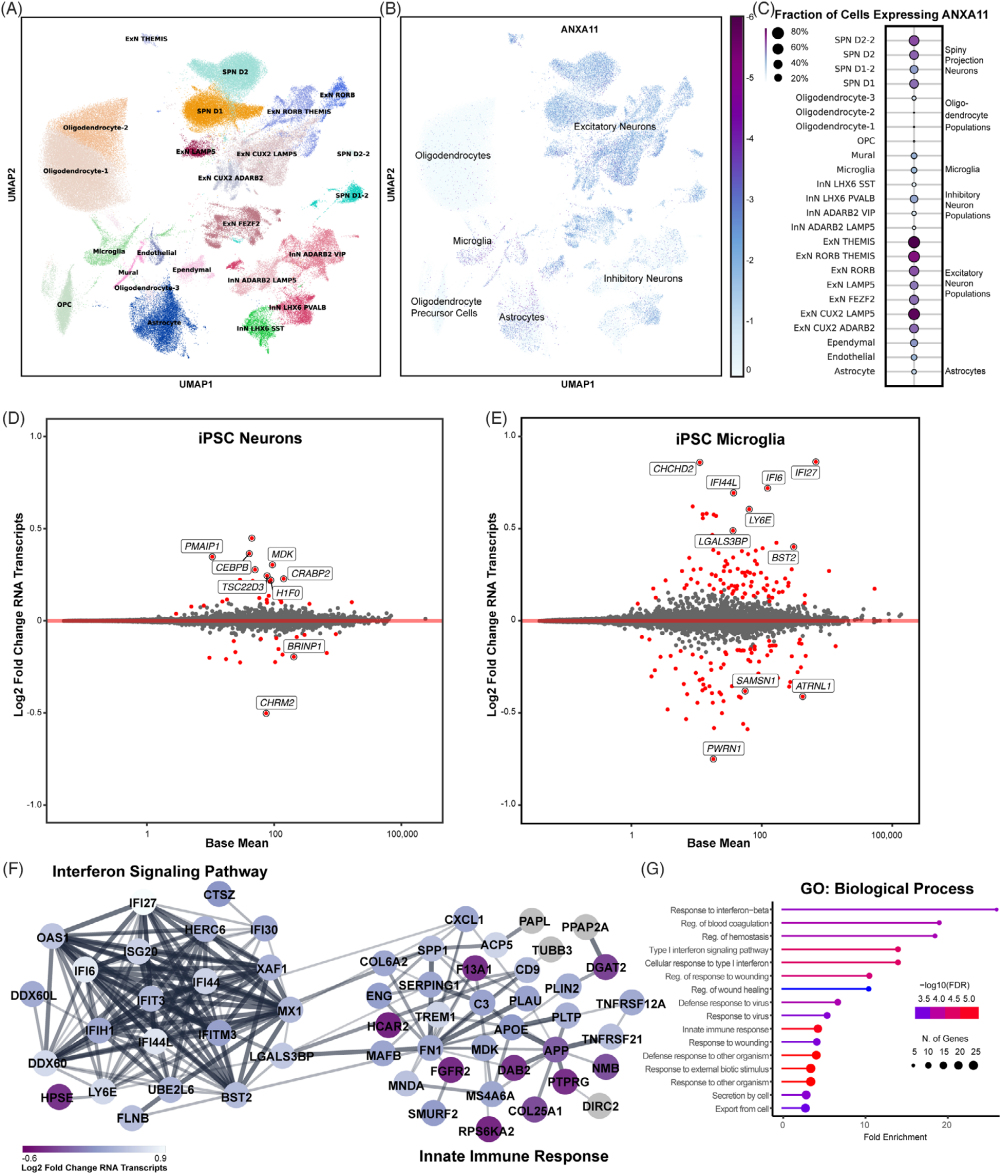
Transcriptomic signature of P93S. (A–C) Expression map of ANXA11 in human brain cells. (A) Single‐nucleus sequencing data from control brains demonstrating the two cell types that highly express ANXA11, microglia, and neurons. Unsupervised clustering of 34 cell types in human brain. UMAP projections illustrating different cell types identified are shown on left (A), and ANXA11 expression levels are shown on right (B). Scale represents log_2_ normalized average gene expression levels. (C) Dot plot illustrating mean normalized expression of ANXA11 by cell type. Scale represents percentage of total cell population expressing ANXA11, with color representing mean normalized expression per cell type. (D and E) Differential gene expression between WT and mutant ANXA11. Bland–Altman mean difference plots where each dot indicates a gene for which there are counted reads from scRNAseq in iPSC‐derived neurons (D) and iPSC‐derived microglia (E). The *x*‐axis is average normalized counts, the *y*‐axis is log_2_ fold change. Neuronal differential expression yields few significant genes related to transcriptional regulation and endocytic vesicles (D) while many more differentially expressed in microglia related to transcriptional regulation and endocytic vesicles (E). (F and G) Differential microglial gene expression interferon signaling pathway. (F) STRING diagram of differentially expressed microglial genes in interferon signaling pathway and innate immune response. Colors indicate *p*_adj_. (G) Gene Ontology terms for biological processes of microglial gene expression hits indicating interferon response and innate immunity. Dot size indicates number of genes in each GO grouping, and color indicates −log_10_(FDR).

Though ANXA11 biology has been most carefully studied in neurons, its role in other cell types in the CNS has not been systematically evaluated. Mining an existing single‐nucleus RNA sequencing dataset of *post mortem* human entorhinal cortex, middle temporal gyrus, putamen, and subventricular zone from cases with no known neurological disease, we found that neurons – in particular, excitatory neurons – and microglia were among the highest ANXA11‐expressing cells in the CNS (Figure 5A‐C).^42^

Next, we profiled transcriptomic and proteomic responses to ANXA11 P93S expression in iPSC‐derived neurons and microglia. We performed transcriptional analysis using single‐cell RNA sequencing. No significant pathways were identified in neuronal transcriptomic results. However, individual review of the differentially expressed genes in the P93S sample primarily related to calcium signaling (eg, *PDE4D*, *RCN1*, and *FSTL5*) cell adhesion and endocytic vesicles (eg, *TENM2*, *CDH13*, *STDN2*, and *CHRM2*) and transcriptional regulation (eg, *SLC3A2*, *PCBD1*, *INSM1, H1F0, EIF1, CRABP2, CEBPB*, and *TSC22D3*) (Figure 5D). Interestingly, microglia exhibited a substantially greater differential gene expression response to ANXA11 P93S expression. GO term analysis revealed alterations in the interferon signaling pathway and innate immune response (Figure 5E‐G).

**FIGURE 6:**
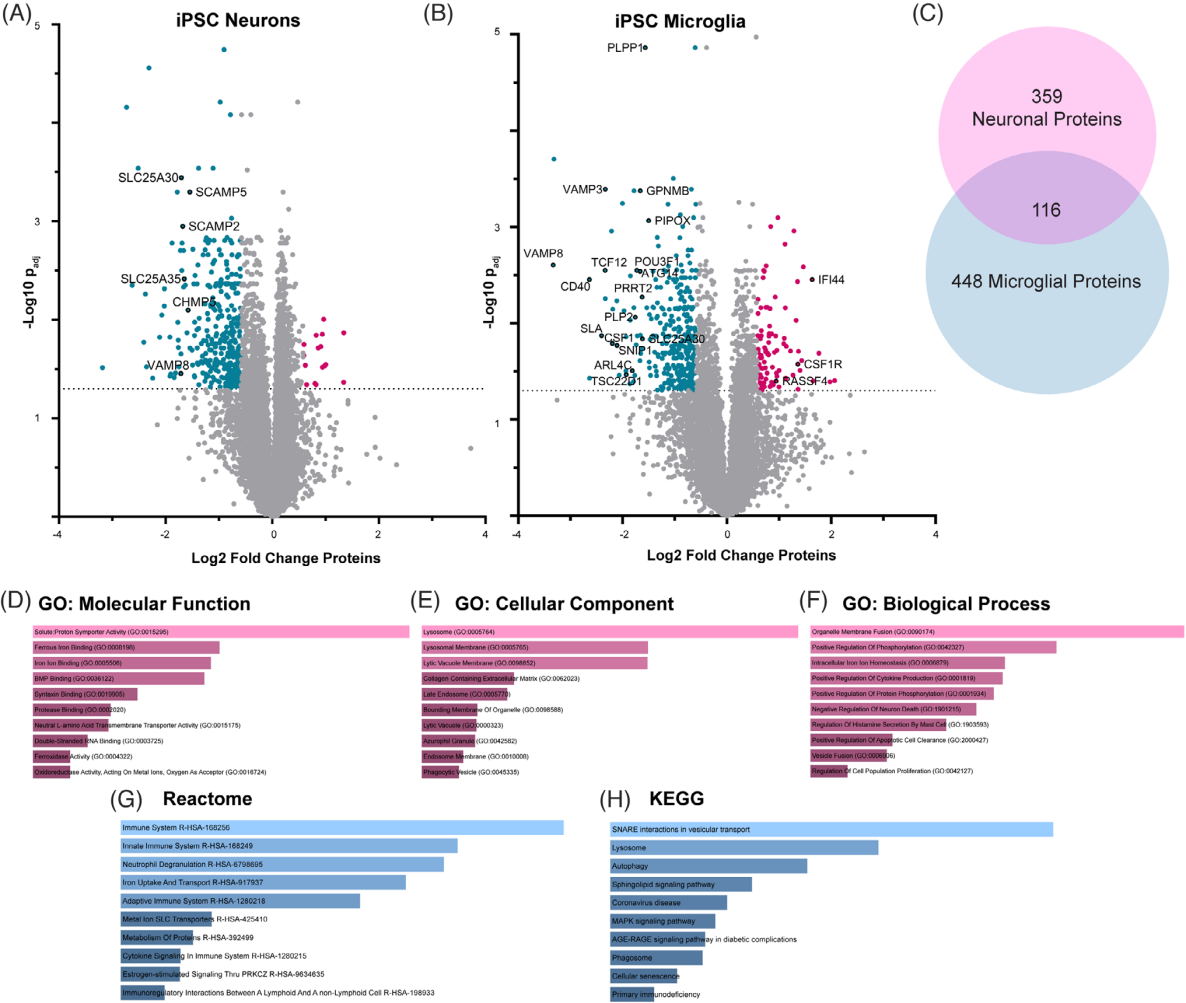
Proteomic signature of P93S. (A) Volcano plot of proteomic changes in neurons identifying significant changes in proteins involved in vesicle transport and SNAREs, corroborating functional assays indicating that P93S disrupts the proper functioning of ANXA11. (B) Volcano plot of proteomic changes of mutant compared to WT microglia implicating inflammatory pathways, as well as transcriptional regulation and vesicular transport. Red dots indicate upregulated proteins (linear FC > 1.5) and blue dots indicate downregulated proteins (linear FC < ‐1.5). The dotted line demarcates a −log_10_ of *p*_adj_ value of 1.3. (C–H) Unique microglia proteomic signature. (C) Venn diagram of significant differentially expressed proteins in neuron (*n* = 359) compared to microglia (*n* = 448). Significance is defined as *p*_adj _> .05. (D–F) Differentially selected microglial peptide gene ontology molecular function (D), cellular component (E), and biological process (F) terms sorted by *p* value with brighter colors indicating more significant values. See Supplementary Material for detailed results. (G and H) Differentially selected microglial peptide reactome (G) and KEGG pathway (H) terms sorted by *p* value, with brighter colors indicating more significant values. See Supplementary File for expanded results.

We further characterized how ANXA11 P93S expression altered the proteome of neurons and microglia using shotgun proteomics. Expression of ANXA11 P93S did not result in any statistically significant increases in protein expression in neurons after correction for multiple comparisons (Figure 6A). However, downregulated neuronal proteins included ribosomal proteins, those involved in vesicle transport, endosome cargo processing, and soluble N‐ethylmaleimide‐sensitive factor attachment protein receptors (SNAREs) (eg, VAMP8, SCAMP2, SLC25A30 and 35, and CHMP5). These proteomic changes in neurons are consistent with functional assays indicating that the P93S mutation disrupts the tethering function of ANXA11.

Proteomic analysis of iPSC‐derived microglia revealed more sizable alterations in the setting of ANXA11 P93S expression (Figure 6A,C). Downregulated proteins broadly grouped into three categories: inflammatory pathways (eg, PLPP1, CD40, SLA, CSF1, GPNMB, PLP2, and PIPOX); transcriptional regulation (eg, SNIP1, POU3F1, TCF12, and TSC22D1); and lysosomal SNAREs involved in vesicular transport (eg, VAMP3, VAMP8, PRRT2, SLC25A30, ATGI4, and ARL4C). Expression of P93S in microglia upregulated proteins associated with the innate immune response, including IFI44 and, to a lesser extent, P2RX4, RBCK1, and SASH1. These findings indicate potential dysregulation of immune pathways, consistent with measured abnormalities in the cellular and protein biomarkers of innate immunity in P93S patient biofluids. Expression of ANXA11 P93S caused greater changes in protein expression in microglia (448 proteins) than in neurons (359 proteins) (Figure 6C). No significant pathways were identified in dysregulated proteins specific to neurons. Dysregulated proteins specific to microglia clustered in lysosome‐related cellular component GO terms (Figure 6E and supplementary file) and biological functions such as cytokine production and vesicle fusion (Figure 6F and supplementary file). Several significant Kyoto Encyclopedia of Genes and Genomes (KEGG) pathways were identified, including those relating to SNARE activity, lysosomes, viral infection, and immune dysregulation (Figure 6H and supplementary file), and several immune system terms and cytokine signaling were identified in reactome analysis (Figure 6G and supplementary file). Taken together, these results reveal that both neurons and microglia may be involved in disease pathophysiology in patients with ANXA11 P93S variants.

## 4 DISCUSSION

This study describes a novel clinical presentation associated with mutations in ANXA11, including the characterization of a previously unreported variant of uncertain significance, and establishes its pathobiology. ANXA11 has been associated with ALS and FTD. More recently, reports expanded the presentation to include an inclusion‐body myopathy‐like multisystem proteinopathy as well as semantic variant primary progressive aphasia.^10^, ^43^, ^44^ We describe a series of cases presenting with CBS, further expanding the phenotypic spectrum of ANXA11 mutations. We use disease‐relevant cellular models to confirm the pathogenicity of the P93S variant through the demonstration of both loss of proper ANXA11 function and pathologic TDP‐43 changes, in addition to identifying cell‐specific bioactivity of the variant through comparative omics.

We previously reported on the function of ANXA11 as a tether between RNA granules and lysosomes during axonal transport, which is disrupted by disease‐causing mutations.^3^ Prolines are unique among amino acids for their cyclical side chain, conferring rigidity in intrinsically disordered domains and solubility to decrease the likelihood of aggregation.^45^, ^46^, ^47^ These properties may be altered with the otherwise relatively conservative amino acid change to a serine. Moreover, amino acid sequences within intrinsically disordered domains, while not necessarily conserved in invertebrates, are highly conserved among vertebrates (Figure 2B,C).^48^, ^49^ Thus, the loss of a proline residue within this region may have an outsized effect on protein structure and function that is not captured by typical in silico prediction models. Despite contradictory annotations using in silico modeling, the specialized function of prolines in LCDs as well as the conserved amino acid sequence in position 93 and the rarity of this variant suggest that the P93S is a deleterious mutation, underscoring the limitations of existing tools to accurately classify VUS. Interrogating the known function of ANXA11 in iPSC‐derived neurons, we showed that this mutation resulted in disrupted tether function and involvement of TDP‐43 pathways, supporting the pathogenicity of this VUS. Therefore, despite conflicting results from in silico predictions, we were able to confirm the pathogenicity of this VUS using cellular models; we demonstrated the utility of these models to inform the classification of variants, especially in disordered protein regions where current predictive models fail.

In neurons, ANXA11 is involved in axonal transport, whereas its role in microglia is not well known. We use unbiased proteomics to detect differential effects of this ANXA11 variant in neurons and microglia. Our cellular models indicate that immune dysregulation is a major affected pathway, and microglia may have an important role in driving pathology. These findings also provide an important link relating to observations in human disease. In our patient biofluids, we find expansion of CD4+ and CD8+ T‐cell populations present in both central and peripheral compartments and elevated CSF IgG, indicating increased blood‐brain barrier permeability and loss of immune system homeostasis. There is growing recognition of the role of inflammation and immune dysregulation in neurodegeneration, including the development of a pro‐inflammatory milieu in the CNS.^50^ Genes implicated in both monogenic neurodegenerative disease and risk genes are related to microglia and immune pathways, such as *APOE*, *TREM2*, and *TBK1*, many of which were also identified in our microglial transcriptomic and proteomic data.^51^, ^52^, ^53^ Like the observations seen in this family, clonal expansion of CD4‐ and CD8‐positive T cells have been described in ALS type four and FTD patients, further supporting a link between immune dysregulation and disease.^54^ Previously published ANXA11 cases reported white matter changes, and we similarly found a unique imaging pattern in this cohort consisting of central predominant atrophy (ie, ex‐vacuo dilatation of the lateral ventricles) in a frontoparietal distribution with T2 FLAIR white matter hyperintensities.^10^ Potential explanations for this include primary oligodendroglial dysfunction, primary axonal injury, or microglial‐based inflammation. However, expression profiling indicates that ANXA11 is not highly expressed in oligodendroglia but is found in microglia. Moreover, white matter has been shown to be particularly vulnerable to inflammation, and microglia are the major immune effector cell within the CNS.^55^ Thus, with the association of ANXA11 with sarcoid, a systemic autoimmune condition, our multiomic findings suggest that microglial ANXA11 contributes to disease and immune dysregulation, reflecting the importance of disease‐relevant cellular models.

LCD sequences are often only conserved among mammals, and specific residues have outsized importance such that even relatively conservative changes can disrupt normal function.^46^, ^48^, ^49^, ^56^ In fact, several established disease‐causing mutations in ANXA11 and other ALS/FTD genes illustrate the limits of in silico modeling for mutations within LCDs (Figure 2D). Accurate variant annotation is critical for its value in understanding the biology as well as the diagnostics and therapeutic implications for patients. The discordant findings from current prediction algorithms highlight the shortcomings of this approach and underscore the need for improved methodology to determine variant pathogenicity. We have demonstrated the feasibility and power of cell‐relevant iPSC‐derived modeling systems to assess the bioactivity of a VUS, search for elements of human disease, and provide evidence of pathogenicity. The identification of a VUS in the context of novel disease phenotypes, such as the one described here, in neurodegenerative disorders with high phenotypic variability poses a particularly vexing challenge. This challenge will only continue to grow with expanded access to genetic testing and widening disease spectra in neurodegeneration, highlighting the critical need for improved methods to classify VUSs.

In conclusion, we describe a family with a novel clinical presentation and a novel VUS in the ALS/FTD‐associated gene ANXA11. Through querying neurodegenerative disorders clinics, we establish CBS as part of the ANXA11 phenotypic spectrum. We also establish the pathogenicity of the P93S VUS by demonstrating decreased colocalization of mutant ANXA11 with lysosomes and a resultant decrease in neuritic RNA in iPSC‐derived neurons, indicating a loss of proper neuronal ANXA11 function. The pathogenicity of this variant is further supported by loss of nuclear TDP‐43 and increased formation of cryptic exons in our cellular model. We establish the multiomic signature of the P93S variant, which supports the functional assay findings in neurons and reveals profound microglial changes related to immune pathways and interferon signaling, in addition to the known role in vesicular transport and transcriptional regulation. This sheds light on the potential role of ANXA11 in microglia and suggests that microglia play an important role in disease pathobiology, relating back to patient observations and human disease. Finally, these findings show the power and promise of using generalizable techniques in cellular models to enhance variant annotation across genes, uncover new genotype–phenotype relationships, and provide insights into disease mechanisms.

## CONFLICT OF INTEREST STATEMENT

Jennifer S. Yokoyama serves on the scientific advisory board for the Epstein Family Alzheimer's Research Collaboration. Debora S. Marks is an advisor for Dyno Therapeutics, Octant, Jura Bio, Tectonic Therapeutic, and Genentech and is a co‐founder of Seismic Therapeutic. The authors Allison Snyder, Veronica H. Ryan, James Hawrot, Sydney Lawton, Daniel M. Ramos, Y. Andy Qi, Kory Johnson, Xylena Reed, Nicholas L. Johnson, Aaron W. Kollasch, Megan Duffy, Lawren VandeVrede, J. Nicholas Cochran, Camilo Toro, Bibiana Bielekova, Justin Y. Kwan, Mark R. Cookson, and Michael E. Ward report no competing interests. Author disclosures are available in the supporting information.

## CONSENT STATEMENT

All human subjects provided informed consent.

## Supporting information

Supporting information

Supporting information

Supporting information

## Data Availability

The data that support the findings of the clinical spectrum of ANXA11 mutations can be found at https://adknowledgeportal.synapse.org/Explore/Studies/DetailsPage/StudyDetails?Study=syn25686496, but further clinical information will not be made publicly available to protect the privacy of research participants. The code for the analysis of single‐cell sequencing can be found at: https://github.com/NIH‐CARD/ANXA11_novel_variant.git, and MS‐based proteomic findings are openly available at PRIDE. The data that support the remaining findings in this study are available from the corresponding author upon request.

## References

[1] Fayer S , Horton C , Dines JN , et al. Closing the gap: systematic integration of multiplexed functional data resolves variants of uncertain significance in BRCA1, TP53, and PTEN. Am Hum Genet. 2021;108:2248‐2258. doi:10.1016/J.AJHG.2021.11.001 PMC8715144 34793697

[2] Costain G , Andrade DM . Third‐generation computational approaches for genetic variant interpretation. Brain. 2023;146:411‐412. doi:10.1093/BRAIN/AWAD011 36691296

[3] Liao YC , Fernandopulle MS , Wang G , et al. RNA granules hitchhike on lysosomes for long‐distance transport, using annexin a11 as a molecular tether. Cell. 2019;179:147‐164.e20. doi:10.1016/J.CELL.2019.08.050 31539493 PMC6890474

[4] Nahm M , Lim SM , Kim YE , et al. ANXA11 mutations in ALS cause dysregulation of calcium homeostasis and stress granule dynamics. Sci Transl Med. 2020;12:566. doi:10.1126/SCITRANSLMED.AAX3993 33087501

[5] Sainouchi M , Hatano Y , Tada M , et al. A novel splicing variant of ANXA11 in a patient with amyotrophic lateral sclerosis: histologic and biochemical features. Acta Neuropathol Commun. 2021;9:106. 34099057 10.1186/s40478-021-01202-w PMC8186038

[6] Lillebostad PAG , Raasakka A , Hjellbrekke SJ , et al. Structure of the ALS mutation target annexin A11 reveals a stabilising N‐terminal segment. Biomolecules. 2020;10:660. doi:10.3390/BIOM10040660 32344647 PMC7226064

[7] Hofmann S , Franke A , Fischer A , et al. Genome‐wide association study identifies ANXA11 as a new susceptibility locus for sarcoidosis. Nat Genet. 2008;40:1103‐1106. doi:10.1038/NG.198 19165924

[8] Topp SD , Smith BN, Fallini C , et al. Mutations in the vesicular trafficking protein annexin A11 are associated with amyotrophic lateral sclerosis. Sci Transl Med. 2017;9:388. doi:10.1126/SCITRANSLMED.AAD9157 PMC6599403 28469040

[9] Zhang K , Liu Q , Liu K , et al. ANXA11 mutations prevail in Chinese ALS patients with and without cognitive dementia. Neurol Genet. 2018;4:3. doi:10.1212/NXG.0000000000000237 PMC5963931 29845112

[10] Leoni TB , González‐Salazar C , Rezende TJR , et al. A novel multisystem proteinopathy caused by a missense ANXA11 variant. Ann Neurol. 2021:90(2):239‐252. doi:10.1002/ana.26136 34048612

[11] Hannikainen PA , Kosa P , Barbour C , Bielekova B . Extensive healthy donor age/gender adjustments and propensity score matching reveal physiology of multiple sclerosis through immunophenotyping. Front Neurol. 2020;11:565957. doi:10.3389/FNEUR.2020.565957/BIBTEX 33329307 PMC7732581

[12] Fernandopulle MS , Prestil R , Grunseich C , Wang C , Gan L , Ward ME . Transcription factor‐mediated differentiation of human iPSCs into neurons. Curr Protoc Cell Biol. 2018;79:e51. doi:10.1002/cpcb.51 29924488 PMC6993937

[13] Dräger NM , Sattler SM , Huang CT‐L , et al. A CRISPRi/a platform in human iPSC‐derived microglia uncovers regulators of disease states. Nat Neurosci. 2022;2022:1‐14. doi:10.1038/s41593-022-01131-4 PMC9448678 35953545

[14] Wang F , Flanagan J , Su N , et al. RNAscope: a novel in situ RNA analysis platform for formalin‐fixed, paraffin‐embedded tissues. J Mol Diagn. 2012;14:22‐29. doi:10.1016/J.JMOLDX.2011.08.002 22166544 PMC3338343

[15] Choi HMT , Schwarzkopf M , Fornace ME , et al. Third‐generation in situ hybridization chain reaction: multiplexed, quantitative, sensitive, versatile, robust. Development (Cambridge). 2018;145. doi:10.1242/DEV.165753/48466 PMC6031405 29945988

[16] Hao Y , Hao S , Andersen‐Nissen E , et al. Integrated analysis of multimodal single‐cell data. Cell. 2021;184:3573‐3587.e29. doi:10.1016/J.CELL.2021.04.048 34062119 PMC8238499

[17] R Core Team . R: A Language and Environment for Statistical Computing. R Foundation for Statistical Computing (Vienna, Austria). 2021. https://www.R‐project.org/

[18] Love MI , Huber W , Anders S . Moderated estimation of fold change and dispersion for RNA‐seq data with DESeq2. Genome Biol. 2014;15:1‐21. doi:10.1186/S13059-014-0550-8/FIGURES/9 PMC4302049 25516281

[19] Ge SX , Jung D , Jung D , Yao R . ShinyGO: a graphical gene‐set enrichment tool for animals and plants. Bioinformatics. 2020;36:2628‐2629. doi:10.1093/BIOINFORMATICS/BTZ931 31882993 PMC7178415

[20] Shannon P , Markiel A , Ozier O , et al. Cytoscape: a software environment for integrated models of biomolecular interaction networks. Genome Res. 2003;13:2498. doi:10.1101/GR.1239303 14597658 PMC403769

[21] Hughes CS , Moggridge S , Müller T , Sorensen PH , Morin GB , Krijgsveld J . Single‐pot, solid‐phase‐enhanced sample preparation for proteomics experiments. Nat Protoc. 2018;14:68‐85. doi:10.1038/s41596-018-0082-x 30464214

[22] Reilly L , Lara E, Ramos DM, et al. A fully automated FAIMS‐DIA mass spectrometry‐based proteomic pipeline. Cell Reports Methods. 2023; 3, 10. doi:10.1016/j.crmeth.2023.100593 PMC10626189 37729920

[23] Xie Z , Bailey A , Kuleshov MV , et al. Gene set knowledge discovery with Enrichr. Curr Protoc. 2021;1:e90. doi:10.1002/CPZ1.90 33780170 PMC8152575

[24] Kuleshov MV , Jones MR , Rouillard AD , et al. Enrichr: a comprehensive gene set enrichment analysis web server 2016 update. Nucleic Acids Res. 2016;44:W90‐W97. doi:10.1093/NAR/GKW377 27141961 PMC4987924

[25] Chen EY , Tan CM , Kou Y , et al. Enrichr: interactive and collaborative HTML5 gene list enrichment analysis tool. BMC Bioinf. 2013;14:1‐14. doi:10.1186/1471-2105-14-128/FIGURES/3 PMC3637064 23586463

[26] Cunningham F , Allen JE , Allen J , et al. Ensembl 2022. Nucleic Acids Res. 2022;50:D988‐D995. doi:10.1093/NAR/GKAB1049 34791404 PMC8728283

[27] Ng PC , Henikoff S . SIFT: predicting amino acid changes that affect protein function. Nucleic Acids Res. 2003;31:3812. doi:10.1093/NAR/GKG509 12824425 PMC168916

[28] Rentzsch P , Witten D , Cooper GM , Shendure J , Kircher M . CADD: predicting the deleteriousness of variants throughout the human genome. Nucleic Acids Res. 2019;47:D886‐D894. doi:10.1093/NAR/GKY1016 30371827 PMC6323892

[29] Ioannidis NM , Rothstein JH , Pejaver V , et al. REVEL: an ensemble method for predicting the pathogenicity of rare missense variants. Am J Hum Genet. 2016;99:877. doi:10.1016/J.AJHG.2016.08.016 27666373 PMC5065685

[30] Adzhubei IA , Schmidt S , Peshkin L , et al. A method and server for predicting damaging missense mutations. Nat Methods. 2010;7:248‐249. doi:10.1038/NMETH0410-248 20354512 PMC2855889

[31] Davydov EV , Goode DL , Sirota M , Cooper GM , Sidow A , Batzoglou S . Identifying a high fraction of the human genome to be under selective constraint using GERP++. PLoS Comput Biol. 2010;6:e1001025. doi:10.1371/JOURNAL.PCBI.1001025 21152010 PMC2996323

[32] Dong C , Wei P , Jian X , et al. Comparison and integration of deleteriousness prediction methods for nonsynonymous SNVs in whole exome sequencing studies. Hum Mol Genet. 2015;24:2125‐2137. doi:10.1093/HMG/DDU733 25552646 PMC4375422

[33] Reva B , Antipin Y , Sander C . Predicting the functional impact of protein mutations: application to cancer genomics. Nucleic Acids Res. 2011;39:e118‐e118. doi:10.1093/NAR/GKR407 21727090 PMC3177186

[34] Frazer J , Notin P , Dias M , et al. Disease variant prediction with deep generative models of evolutionary data. Nature. 2021;599(7883):91‐95. doi:10.1038/s41586-021-04043-8 34707284

[35] Shir D , Pham NTT , Botha H , et al. Clinicoradiologic and neuropathologic evaluation of corticobasal syndrome. Neurology. 2023;101:E289‐E299. doi:10.1212/WNL.0000000000207397 37268436 PMC10382268

[36] Neumann M , Sampathu DM , Kwong LK , et al. Ubiquitinated TDP‐43 in frontotemporal lobar degeneration and amyotrophic lateral sclerosis. Science. 2006;314:130‐133. doi:10.1126/SCIENCE.1134108 17023659

[37] Ling JP , Pletnikova O , Troncoso JC , Wong PC . TDP‐43 repression of nonconserved cryptic exons is compromised in ALS‐FTD. Science. 2015;349:650‐655. doi:10.1126/SCIENCE.AAB0983 26250685 PMC4825810

[38] Klim JR , Williams LA , Limone F , et al. ALS‐implicated protein TDP‐43 sustains levels of STMN2, a mediator of motor neuron growth and repair. Nat Neurosci. 2019;22:167‐179. doi:10.1038/s41593-018-0300-4 30643292 PMC7153761

[39] Melamed Z , López‐Erauskin J , Baughn MW , et al. Premature polyadenylation‐mediated loss of stathmin‐2 is a hallmark of TDP‐43‐dependent neurodegeneration. Nat Neurosci. 2019;22:180‐190. doi:10.1038/S41593-018-0293-Z 30643298 PMC6348009

[40] Ma XR , Prudencio M , Koike Y , et al. TDP‐43 represses cryptic exon inclusion in the FTD–ALS gene UNC13A. Nature. 2022;603(7899):124‐130. doi:10.1038/s41586-022-04424-7 35197626 PMC8891019

[41] Brown AL , Wilkins OG , Keuss MJ , et al. TDP‐43 loss and ALS‐risk SNPs drive mis‐splicing and depletion of UNC13A. Nature. 2022;603(7899):131‐137. doi:10.1038/s41586-022-04436-3 35197628 PMC8891020

[42] Duffy MF , Ding J , Langston RG , et al. Divergent patterns of healthy aging across human brain regions at single‐cell resolution reveal links to neurodegenerative disease. Biorxiv. 2023:2023.07.31.551097. doi:10.1101/2023.07.31.551097

[43] Kim EJ , Moon SY , Kim HJ , Jung NY , Lee SM , Kim YE . Semantic variant primary progressive aphasia with a pathogenic variant p.Asp40Gly in the ANXA11 gene. Eur J Neurol. 2022;29:3124‐3126. doi:10.1111/ENE.15455 36073198

[44] Johari M , Papadimas G , Papadopoulos C , et al. Adult‐onset dominant muscular dystrophy in Greek families caused by Annexin A11. Ann Clin Transl Neurol. 2022;9(10):1660‐1667. doi:10.1002/ACN3.51665 36134701 PMC9539373

[45] Theillet F‐X , Kalmar L , Tompa P , et al. The alphabet of intrinsic disorder: I. Act like a Pro: on the abundance and roles of proline residues in intrinsically disordered proteins. Intrinsically Disord Proteins. 2013;1:e24360. doi:10.4161/IDP.24360 28516008 PMC5424786

[46] Darling AL , Liu Y , Oldfield CJ , Uversky VN . Intrinsically disordered proteome of human membrane‐less organelles. Proteomics. 2018;18:1700193. doi:10.1002/PMIC.201700193 29068531

[47] Wang J , Choi JM , Holehouse AS , et al. A molecular grammar governing the driving forces for phase separation of prion‐like RNA binding proteins. Cell. 2018;174:688‐699.e16. doi:10.1016/j.cell.2018.06.006 29961577 PMC6063760

[48] Nido GS , Méndez R , Pascual‐García A , Abia D , Bastolla U . Protein disorder in the centrosome correlates with complexity in cell types number. Mol Biosyst. 2012;8:353‐367. doi:10.1039/C1MB05199G 22076659

[49] Kulkarni P , Uversky VN . Intrinsically disordered proteins: the dark horse of the dark proteome. Proteomics. 2018;18:1800061. doi:10.1002/PMIC.201800061 30218496

[50] Snyder A , Grant H , Chou A , et al. Immune cell counts in cerebrospinal fluid predict cognitive function in aging and neurodegenerative disease. Alzheimers Dement. 2023;19:3339‐3349. doi:10.1002/ALZ.12956 36791265 PMC10425564

[51] Sirkis DW , Bonham LW , Yokoyama JS . The role of microglia in inherited white‐matter disorders and connections to frontotemporal dementia. Appl Clin Genet. 2021;14:195‐207. doi:10.2147/TACG.S245029 33833548 PMC8020808

[52] Bonham LW , Sirkis DW , Yokoyama JS . The transcriptional landscape of microglial genes in aging and neurodegenerative disease. Front Immunol. 2019;10:1170. doi:10.3389/fimmu.2019.01170 31214167 PMC6557985

[53] Sawyer RP , Hill EJ , Yokoyama J , et al. Differences in peripheral immune system gene expression in frontotemporal degeneration. Medicine (Baltimore). 2022;101:e28645. doi:10.1097/MD.0000000000028645 35060553 PMC8772666

[54] Campisi L , Chizari S , Ho JSY , et al. Clonally expanded CD8 T cells characterize amyotrophic lateral sclerosis‐4. Nature. 2022;606:945‐952. doi:10.1038/S41586-022-04844-5 35732742 PMC10089623

[55] Raj D , Yin Z , Breur M , et al. Increased white matter inflammation in aging‐ and Alzheimer's disease brain. Front Mol Neurosci. 2017;10:206. doi:10.3389/FNMOL.2017.00206/FULL 28713239 PMC5492660

[56] King OD , Gitler AD , Shorter J . The tip of the iceberg: RNA‐binding proteins with prion‐like domains in neurodegenerative disease. Brain Res. 2012;1462:61‐80. doi:10.1016/J.BRAINRES.2012.01.016 22445064 PMC3372647

