# Supplementary material for: An ANXA11 P93S variant dysregulates TDP‐43 and causes corticobasal syndrome
Source: Alzheimers Dement. 2024 Jun 26;20(8):5220–35. doi: 10.1002/alz.13915 (PMC11350008; doi:10.1002/alz.13915)
Supplement: Supplementary file 1 — Supporting information [file ALZ-20-5220-s001.docx]

**Supplementary material**

**Cryptic Exon Detection Probe Sequences**

**STMN2 native HCR FISH probe sequences:** TCACTgATCTgCTCTTgCTTTTACCCggAACCTCgCAACATCAACATCTATA

TACgATgATATggAAgTgAAgCAAATCAACAAACgTgCCTCTggCCAggCTT

gAgCTgATCTTgAAgCCACCATCTCCTATCTCAgAAgCCCCACgAACTTTAg

TTCAgCAAgATggCggAggAAAAgCTgATCCTgAAAATggAACAAATTAAgg

CATgCTgCggAggTgCgCAggAACAAggAACTCCAggTTgAACTgTCTggCT

**STMN2 cryptic HCR FISH probe sequences**:

gACTCggCAgAAgACCTTCgAgAgAAAggTAgAAAATAAgAATTTggCTCTC

TgTgAgCATgTgTgCgTgTgTgCgAgAgAgAgAgACAgACAgCCTgCCTAAg

gAAATgAATgTgAATgCggCTTgTggCACAgTTgACAAggATgATAAATCAA

**UNC13A native HCR FISH probe sequences:**

CgTCTggATTTgggACTgACggTggAggTgTggAATAAgggTCTCATCTggg

gAgCTggAggAggAggAggAggAggTgCCTgACgATTTgggCAgCTATgCCC

CgTgAAgACgTAgCTgTggCTgAgCCCAAAgACTTCAAACgCATCAgCCTCC

TTTgAgCTACCCTTAgACATTCCTgAAgAggAggCTCgCTACTgggCCAAgA

CTggAgCAgCTCAATgCTATgCgggACCAggATgAATATTCgTTCCAAgATg

ggCTggggTgAgCAgCACAACgATgACCCCgACAgTgCAgTggATgATCgTg

TCACAACCCAACgCCTCAgTCCACCAATATTCTgTTCgCCCACCACCCCTgg

TCCCgggAgTCCTACAgTgACTCCATgCACAgTTACgAggAgTTCTCTgAgC

CAAgCCCTCAgCCCCACgggTAgCAgCCgCTATgCCTCTTCCggggAgCTgA

CAgggAAgCTCTCAgCTgAgCgAggACTTCgACCCTgACgAgCACAgCCTgC

gAggAggACCTggAggACTTCCTggAggAggAggAgCTgCCTgAAgATgAgg

gAgggCCCTggAgAgTggCTgACgCTggACTCCCAggTCATCATggCAgACA

CAAgACAAgCCTCTgCCTgTCCCCAgCAACCAgTgCTgCAACTggAATTATT

AgTgACTACCgCAgTgAAACgAgCAACAgCATCCCgCCgCCCTATTATACTA

ggCTCCgACATggAggATgAgCgggACCgggACTCCTACCACTCCTgCCACA

gACATggCCAAggTggCCCCCAAgCCAgCCACgCCCgACAAggTgCCTgCAg

gAgCAgATCCCTgAggCTgAgCCACCCAAggACgAggAgAgTTTCAggCCgA

gAggATgAggAAggCCAggAggggCAggACTCCATgTCCAgggCCAAggCCA

ATCATCgACAgCATgCCAgACATCCgCAAgAggAAACCTATCCCACTCgTgA

gCCTCCAgCACgTTgAACAACgAggAgCTgAAAAACCACgTTTACAAgAAgA

**UNC13A cryptic HCR FISH probe sequences**:

CTgCCTgggTTTCCTggAAAgAACTCTTATCCCCAggAACTAgTTTgTTgAA

AATgCTggTgAATgAATgAATgATTgAACAgATgAATgAgTgATgAgTAgAT

**Expanded Clinical Descriptions**

**Patient 1:**

The proband developed her initial symptom of bradykinesia and right leg spasticity at age 60. She had a slowly progressive disease course characterized by asymmetric spasticity, dystonia, and apraxia. Late in her disease course she developed dysarthria and dysphagia and ultimately became mute. Her symptoms evolved over the course of 15 years. At the time of her clinical evaluation, patient was mute and quadriparetic therefore detailed testing of praxis and cortical sensations could not be assessed. EMG/NCS revealed no fibrillations or sharp waves and was without evidence of active denervation. Her MR imaging revealed atrophy and progressive periventricular white matter hyperintensities. Spinal fluid analysis revealed elevated protein and IgG and IgG index with negative oligoclonal bands. CSF immunoprofiling demonstrated elevated proportions of innate lymphoid cells and higher proportion of CSF granulocytes, as well as elevated NFL, a marker of neuro-axonal injury, and CHIT3L1 a marker associated with intrathecal activation of innate immunity, expressed mainly in astrocytes and macrophages/microglia. She died at age 77 due to aspiration pneumonia.

**Patient 2:**

The proband’s second oldest brother developed symptoms of asymmetric lower extremity onset spasticity beginning around age 50 and progressing to apraxia with dystonia, cortical sensory loss, and alien limb slowly over the course of more than twenty years. EMG/NCS demonstrated an L5/S1 radiculopathy in addition to short duration motor unit potentials and early recruitment pattern in the iliopsoas muscle. His MR imaging revealed extensive periventricular and juxtacortical white matter lesions supratentorially as well as in the brainstem, cervical and thoracic spine. There was significant central atrophy and thinning of the corpus callosum. Cerebrospinal fluid analysis revealed elevated protein and IgG, without oligoclonal bands. Immunoprofiling demonstrated elevated proportions of innate lymphoid cells in blood and highly elevated CSF NFL, a marker of neuro-axonal injury, and CHIT3L1 a marker associated with intrathecal activation of innate immunity, expressed mainly in astrocytes and macrophages/microglia. The patient remains alive with his symptoms at age 79.

**Patient 3:**

The proband’s oldest brother died at age 49 after a slowly progressive course with asymmetric lower extremity onset inability to walk, tremor, and dysarthria. He additionally was described as having agitation and irritability. He carried a diagnosis of primary progressive multiple sclerosis and the family was told that he had a “white” lesions in his brain.

**Patient 4:**

The proband’s father died around age 60 after having developed gait dysfunction described as dragging his feet, imbalance and incoordination, dysarthria and dysphagia and dystonic faces.

**Patients 5-9:**

Additional cases include a 66-year-old male with the G189E mutation with fifteen years of progressive asymmetric lower extremity onset spasticity, dysarthria, and dysphagia, elevated IgG in the CSF and matched oligoclonal bands as well as white matter lesions on MRI. A patient carrying the Y103H VUS presented with left arm apraxia and dystonia beginning at age 59 and posterior predominant atrophy on MRI with a notched corpus callosum, diagnosed clinically with corticobasal syndrome. A patient carrying the R404W VUS demonstrated posterior centrally predominant atrophy, thinning of the corpus callosum and white matter hyperintensities on MR was diagnosed with corticobasal syndrome. A patient with the S55L VUS was diagnosed with behavioral variant FTD and was notable for having a prolonged disease course, parkinsonism, and an abnormal gait with an MRI showing extreme central atrophy. This S55L individual had a brother reported to have progressive spasticity, cortical sensory loss and dysphagia over more than a decade consistent with corticobasal syndrome, although no formal diagnosis was made.

**
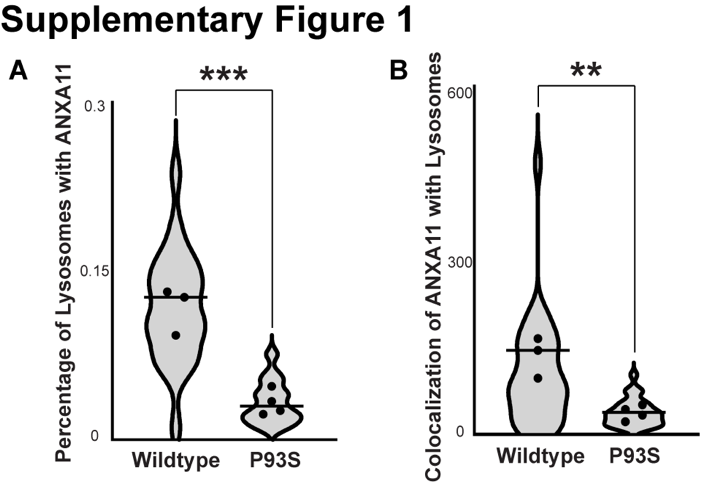
**

**Supplemental Figure 1 Decreased colocalization of lysosomes in mutant ANXA11 neurons**. **(A)** Quantification of percent of total lysosomes with ANXA11, well mean indicated by dot, horizontal line indicates median, p = 0.0009. **(B)** Quantification of number of ANXA11 puncta colocalized with lysosomes, well mean indicated by dot, horizontal line indicates median, p = 0.0031.

**
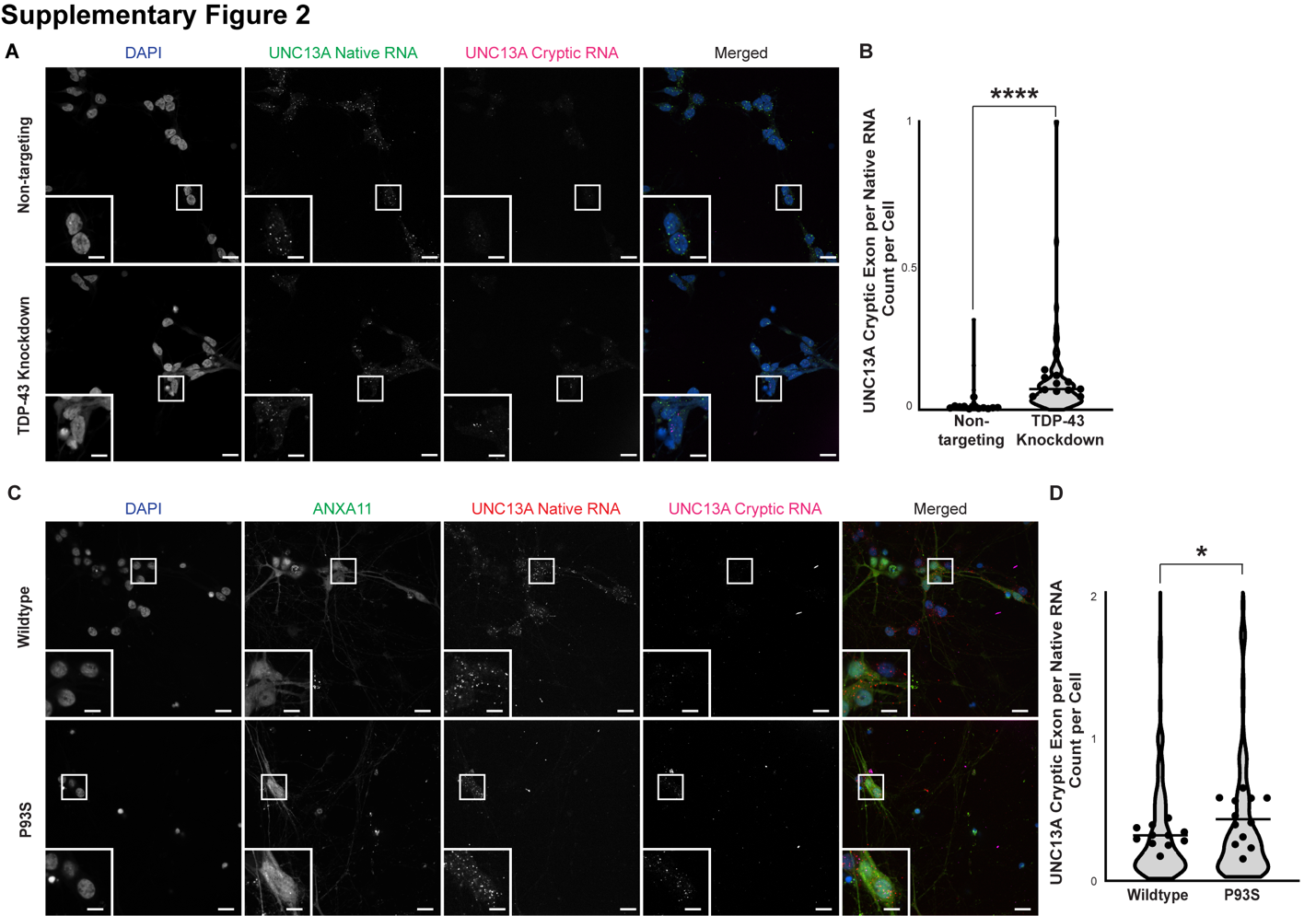
**

**Supplemental Figure 2 Increased UNC13A cryptic exon formation in mutant ANXA11 neurons. (A)** Representative images of fixed iPSC-derived CRISPRi neurons with control non-targeting and TDP-43 knockdown guides demonstrating detection of native UNC13A RNA (green) and cryptic RNA (magenta) using HCR FISH probes with Hoechst nuclear counterstaining (blue), scale bar = 25 µm, inset scale bar = 4 µm. **(B)** Quantification of the ratio of cryptic RNA per native per cell in non-targeting and TDP-43 knockdown cells for UNC13A, well mean indicated by dot, horizontal line indicates median, Mann Whitney p<0.0001. **(C)** Representative images of fixed iPSC-derived neurons expressing wildtype and mutant ANXA11 (green) with HCR FISH probes for native STMN2 RNA (red) and cryptic RNA (magenta) with Hoechst nuclear counterstaining (blue), scale bar = 25 µm, inset scale bar = 4 µm. **(D)** Quantification of the ratio of cryptic RNA per native per cell in wildtype and mutant ANXA11 cells for UNC13A, well mean indicated by dot, horizontal line indicates median, p = 0.04.
